# High Species Diversity of the Leafhopper Genus *Hishimonus* Ishihara (Hemiptera: Cicadellidae: Deltocephalinae) from China, with Description of Ten New Species

**DOI:** 10.3390/insects10050120

**Published:** 2019-04-26

**Authors:** Lan Du, Wu Dai

**Affiliations:** Key Laboratory of Plant Protection Resources and Pest Management, Ministry of Education, Entomological Museum, Northwest A&F University, Yangling, Shaanxi 712100, China; 15848994911@163.com

**Keywords:** Opsiini, morphology, taxonomy, distribution, China

## Abstract

*Hishimonus* Ishihara is a large genus of leafhoppers widely distributed in Asia, Africa, and Australia. Its greatest diversity appears to be in the Oriental region where recent investigations on *Hishimonus* suggest much of the diversity remains undocumented. In the present study, twenty-eight species of the leafhopper genus *Hishimonus* Ishihara from China are reviewed based on comparative morphological study, including ten new species: *H. fletcheri* sp. nov., *H. lii* sp. nov., *H. dietrichi* sp. nov., *H. kuohi* sp. nov., *H. tenuis* sp. nov., *H. hamuleus* sp. nov., *H. triangulus* sp. nov., *H. virakatmathiellus* sp. nov., *H. paraphycitis* sp. nov., and *H. yuanmouensis* sp. nov. Two additional species (*Hishimonus knightiellus* Virktamath & Murthy and *Hishimonus subtilis* Knight) are recorded from China for the first time. Furthermore, three new synonymies are proposed: *Hishimonus reflexus* Kuoh, 1976 syn. nov. and *Hishimonus biuncinatus* Li, 1988 syn. nov. are reduced to synonymy with *Hishimonus bucephalus* Emeljanov, 1969; *Hishimonus lamellatus* Cai & Kuoh, 1995 syn. nov. is a junior synonym of *Hishimonus expansivus* Li, 1988. All the taxa are described and illustrated based on observations of specimens from China. The first study of the female genitalia of Chinese *Hishimonus* reveals considerable similarity among the *Hishimonus* species studied. A key and list of all Chinese *Hishimonus* species are also provided.

## 1. Introduction

China is one of the world’s most biodiverse countries, hosting more than ten percent of the world’s known species [1], including a considerable number of endemic species. The rich diversity of insect taxa may be explained in part by the country’s complicated geological history and dramatic variation in local climates. Therefore, insect diversity research and conservation efforts in China are necessary.

Leafhoppers are phytophagous insects in the order Hemiptera and constitute the largest hemipteran family, with more than 22,000 species. However, recent investigations on leafhoppers in China suggest that many species remain undocumented [2,3,4,5]. *Hishimonus* is a large and economically important genus, comprising 62 described species, distributed exclusively in the temperate and subtropical regions of the Old World. Recent reviews of the species of the genus *Hishimonus* Ishihara from Australia [6], Thailand [7], and the Indian subcontinent [8] have resulted in a better understanding of the distribution of the species of this genus in the Oriental and Australian regions, but the fauna of China remains understudied.

In his revision of the genus, Knight [9] suggested that certain species may be endemic to a particular region, but later Dai et al. [7] showed that some of these species are more widely distributed. Because some species of *Hishimonus* have been shown to transmit pathogens to economically important plants, reliable specimen identification and biodiversity monitoring of *Hishimonus* in the field is necessary and requires development of improved identification tools.

Li et al. [10] dealt with 14 species of *Hishimonus* out of 17 known from China. One of their species, *Hishimonus tripunctatus* Li, 2011, was transferred to *Litura* Knight by Viraktamath & Murthy [8]. Our re-examination of some of the species described by Kuoh [11], Li [12], and Li & Wang [13] revealed the need for a comprehensive review of the species of *Hishimonus* from China.

In our study, we first assessed *Hishimonus* diversity in China through field surveys, particularly during three targeted expeditions in 2012–2013. The opportunity is taken here to redescribe the known species of *Hishimonus* from China and update nomenclature. Ten new species discovered are described here. All the taxa treated are illustrated, and a key to known species of *Hishimonus* from China is also provided.

## 2. Materials and Methods

The materials examined were collected from several provinces in China and deposited in the Entomological Museum of Northwest A&F University (NWAFU), Yangling, Shaanxi, China.

Abdomens were removed from specimens and soaked in 10% KOH for 24 h to dissolve the muscle, washed in distilled water, and then transferred to glycerin for further dissection and examination. Specimen micrographs were taken using OLYMPUS PM-10AD and Nikon AFX-II stereomicroscope with a Q Imaging digital camera, captured in Q-Capture Pro 7 (SciTech P/L) and compiled in Auto-Montage Pro (Synoptics Ltd, Cambridge, UK). The resulting images were improved and prepared as plates using Adobe Photoshop CS2.

Morphological terminology followed Dai et al. [7] and Viraktamath & Murthy [8].

## 3. Results and Discussion

### 3.1. Generic Character

#### *Hishimonus* Ishihara, 1953

##### *Hishimonus* Ishihara, 1953: 38. Type Species: *Thamnotettix sellata* Uhler, 1896

**Description.** (Modified from Knight 1970 [9], Dai et al. 2013 [7] and Viraktamath & Murthy 2014 [8]).

Small species, 3.0–5.0 mm in length. Coloration usually greenish or yellow with legs stramineous, usually with symmetrical brown markings dorsally on head, pronotum, and forewing, forewings silvery white with dark brown mottling, and usually with a large brown semicircular spot at midlength of commissural margin forming, when the wings at rest, a conspicuous median diamond-shaped spot with that of opposite wing.

Head subequal to or wider than pronotum, slightly longer medially than next to eye with anterior margin broadly rounded; narrowly rounded to face; frontoclypeus longer than wide with lateral margins expended; anteclypeus elongate with lateral margins parallel over basal two thirds, slightly expanded at apex; genal margins concave beneath eyes; ocelli on anterior margin, contiguous with eyes or separated by a distance less than its own diameter; a shallow transverse furrow on vertex between ocelli; vertex posterior to furrow glabrous, face, and vertex anterior to furrow shagreened. Pronotum approximately twice the length of vertex, sides short, posterior margin transverse or shallowly concave; surface glabrous. Scutellum triangular, wider than long, slightly shorter than pronotum. Forewings with claval veins joined by cross vein at midlength; inner subapical cell open basally; outer subapical cell elongate with ends transverse; appendix well developed. Fore femur with setal arrangement as follows: 1 AD and 1 PD at apex, AM1 well developed, 9–13 IC, 6–8 PV and 12–13 short AV. Fore tibia with 1 + 4 (AD + PD) setae. Apex of hind femora with setal formula 2 + 2 + 1.

Pygofer usually sclerotized, lobe acutely rounded posteriorly with macrosetae over posterior half, without appendage. Segment X well sclerotized dorsoapically and laterally with distinct articulation to dorsal margin of pygofer. Valve large, triangular, posterior margin acutely rounded. Subgenital plates movably articulated to valve, each plate broad and prominent convex basally, gradually or abruptly tapering posteriorly to finger-like process weakly sclerotized, with uniseriate row of stout setae laterally, becoming multiseriate basally, with more lateral rows of long filamentous setae over apical half. Style broadly bilobed basally, median anterior arm prominent extended dorsally to meet connective; preapical lobe pronounced or undeveloped, with several long fine setae; apophysis long, gracile, with minute texturing. Connective Y-shaped with stem long or short. Aedeagus with two dorsally directed shafts each with gonopore near apex, with or without basal processes, usually with apical or preapical processes on shaft.

Female abdominal sternite VII, in ventral view, covering base of ovipositor, broader than long, caudal margin slight or deep concave medially with a process emarginated or not in middle. Female pygofer cover with many macrosetae around posterior margin. First valvulae, in lateral view, distinctly curved dorsally and with dorsal margin concave, with dorsal sculpture concatenate, ventroapical sculpture irregularly reticulate. Second valvulae, in lateral view, with distal toothed blades occupying approximately three-fifths of total length, dorsal margin arcuate with several large teeth distributed over most of blade. Third valvulae, in lateral view, with basal half narrow and apical half distinctly expanded, apex prominently produced roundly, with sparse small setae ventrally.

**Distribution.** China, Afghanistan, Armenia, Australian, Borneo, Ethiopia, Georgia, India, Indonesia, Japan, Korea, Malaysia, Pakistan, Papua New Guinea, Philippines, Russia, Singapore, Slovenia, Sri Lanka, Tanzania, Thailand, United Arab Emirates, Iran, and Oman.

**Notes.** The genus *Hishimonus* belongs to the tribe Opsiini of Deltocephalinae, which is an economically important group of insects because of its ability to cause direct damage to plants and also transmit plant pathogens. Except for the bifurcate aedeagus with a pair of gonopores [14], most species of *Hishimonus* also show an obvious feature in external appearance: a large semicircular brown spot on the forewings, forming a diamond-shaped spot when the wings are at rest. Although this feature is shared with related genera (e.g., *Hishimonoides*, *Litura* and *Naevus* [9]), and Singh [15] suppressed *Hishimonus* Ishihara and *Hishimonoides* Ishihara as generic synonyms of *Cestius* Distant, *Hishimonus* differs in having neither preatrial processes of the aedeagus (sometimes referred to as paraphyses) nor a ventrally extended preatrium without processes [8]. Knight [16] listed the differences between *Cestius* and the *Hishimonus* group (*Hishimonus*, *Hishimonoides*, *Litura*, and *Naevus*), and restored these genera.

Ishihara [17] established *Hishimonus* with *Acocephalus discigutta* Walker, 1857 as the type species and listed *Thamnotettix sellata* Uhler, 1896 as a synonym. However, the status of *A. discigutta* and *T. sellata* have long been controversial. Matsumura [18] and Melichar [19] believed that both are valid species, but, later, Matsumura [20] proposed that *Eutettix sellata* is a synonym of *Eutettix discigutta* and was followed by Distant [21], Merino [22], Esaki & Ito [23], and even Ishihara [17]. After researching material from Thailand, Ishihara [24] corrected his earlier classification and reinstated *Thamnotettix sellata* as a distinct species. Knight [9] examined Walker’s original type material of *A. discigutta* and demonstrated that Ishihara [17] misidentified the species. Thus, the type species of *Hishimonus* is *T. sellata*, which Ishihara [17] originally misidentified as *Hishimonus discigutta*. Nielson [25], Knight [9], and Okada [26] also examined a cotype of *T. sellata* and redescribed it. Ishihara realized his mistake and used *T. sellatus* as the name of the type species in subsequent publications.

Comparative study of the male genitalia of available specimens revealed considerable variation in several structures that appeared to be diagnostic at the species level. The overall structure of the genital capsule was remarkably uniform, but variation occurred in the shape of the caudal margin of the pygofer and the length. The structure of the styles and connective were relatively constant among species with only minor variation in relative proportions and in the shape of the style apophysis and preapical lobe. The aedeagus was also conservative in structure; the main variation occurred in the relative lengths and the shape of shaft, which beared posteriorly directed processes at base in a few species. Species may be divided into two groups based on the structure of the aedeagus. One group had the aedeagus normally developed with the basal processes extended posteriorly. The other group had the aedeagus without basal processes. Variation also occurred in the shape of female sternite VII and the shape and dentition of the female second valvulae.

Some species of *Hishimonus* are known to be of economic importance because of their transmission of plant pathogens such as phytoplasmas: *Hishimonus sellatus* vectors jujube witches’ broom, Mulberry dwarf [27], *Crptotaenia japonica* witches’ broom and Onion yellows [28], Rhus Yellows, and Hovenia witches’ broom [29]; *Hishimonus phycitis* transmits lime witches’ broom [30], sesame phyllody phytoplasma [31], and brinjal little-leaf phytoplasma [32,33]; *Hishimonus concavus* Knight transmits loofah witches’ broom [34].

*Hishimonus* Ishihara is a moderately large genus of 62 species, species of which are predominantly distributed in the Oriental region but with minor extensions into the Ethiopian, Australian, and Palaearctic regions [7,9,35]. Although Knight [9] stated that many of the species they treated were endemic to the areas from which their specimens had originated, in most cases, the material they used was fairly sparse, and there was little evidence presented for endemicity. Dai et al. [7] demonstrated that some species were more widely distributed than original material implied. Recently, a number of additional species have subsequently been added to the genus [6,7,8,36,37], and several species were first recorded from Russia [38], Slovenia [39], Thailand [7], and Pakistan [40], respectively. Nevertheless, many regions or countries where the genus likely occurs still have no records, or there are only scattered records from the earlier published papers or data gathered during occasional collecting. More detailed investigations with expanded taxonomic sampling are needed to address longstanding questions pertaining to species distribution.

##### Checklist of the genus *Hishimonus* from China

*Hishimonus aberrans* Knight, 1970

*Hishimonus bucephalus* Emeljanov, 1969

   *Hishimonus reflexus* Kuoh, 1976 syn. nov.

   *Hishimonus biuncinatus* Li, 1988 syn. nov.

*Hishimonus concavus* Knight, 1970

*Hishimonus dichotomous* Kuoh, 1976

*Hishimonus diffractus* Dai, Fletcher & Zhang, 2013

*Hishimonus expansivus* Li, 1988

   *Hishimonus lamellatus* Cai & Kuoh, 1995 syn. nov.

*Hishimonus fuscomaculatus* Li & Wang, 2004

*Hishimonus hamatus* Kuoh, 1976

*Hishimonus knightiella* Virktamath & Murthy, 2014

*Hishimonus phycitis* (Distant, 1908)

*Hishimonus prolongatus* Li & Wang, 2004

*Hishimonus rectus* Kuoh, 1976

*Hishimonus sellatus* (Uhler, 1896)

*Hishimonus spiniferous* Kuoh, 1976

*Hishimonus subtilis* Knight, 1970

*Hishimonus tortuosus* Kuoh, 1976

*Hishimonus truncatus* Kuoh, 1976

*Hishimonus ventralis* Cai & He, 2001

*Hishimonus tenuis* Du & Dai, sp. nov.

*Hishimonus hamuleus* Du & Dai, sp. nov.

*Hishimonus triangulus* Du & Dai, sp. nov.

*Hishimonus fletcheri* Du & Dai, sp. nov.

*Hishimonus lii* Du & Dai, sp.nov.

*Hishimonus dietrichi* Du & Dai, sp. nov.

*Hishimonus kuohi* Du & Dai, sp.nov.

*Hishimonusviraktamathiellus* Du & Dai, sp. nov.

*Hishimonus paraphycitis* Du & Dai, sp. nov.

*Hishimonus yuanmouensis* Du & Dai, sp. nov.

##### Key to species of *Hishimonus* from China (Figure 1, Figure 2, Figure 3, Figure 4, Figure 5, Figure 6, Figure 7, Figure 8, Figure 9, Figure 10, Figure 11, Figure 12, Figure 13, Figure 14, Figure 15, Figure 16, Figure 17, Figure 18, Figure 19, Figure 20, Figure 21, Figure 22, Figure 23, Figure 24, Figure 25 and Figure 26)

1. Aedeagus with one pair of basal processes…………………………………………………………………………………………………2

- Aedeagus without basal processes……………………………………………………………………………………………………………9

2. Aedeagus with basal process about as long as shaft (Figure 17D)………………………………………………………………………*tortuosus* Kuoh

- Aedeagus with basal process less than one half as long as shaft…………………………………………………………………………3

3. Aedeagus with basal process fused to shaft at base………………………………………………………………………………………7

- Aedeagus with basal process not fused with base of shaft…………………………………………………………………………………4

4. Aedeagal shaft apical hook twice or longer than width of shaft (Figure 17A)…………………………………………………………*prolongatus* Li & Wang

- Aedeagal shaft apical hook not more than 1.5 times as long as shaft width………….…………………………………………………5

5. Aedeagal shaft in lateral view tapered distally, basal process close to shaft, not divergent (Figure 20E)………………………*aberrans* Knight

- Aedeagal shaft in lateral view of uniform width; basal process divergent from shaft…………………………………………………6

6. Aedeagal shaft in lateral view straight distally (Figure 20C)……………………………………………………………………………*kuohi* sp. nov.

- Aedeagal shaft in lateral view arched distally (Figure 20B)………………………………………………………………………………*spiniferous* Kuoh

7. Aedeagus basal process anterior to shaft; each process curved posteriorly in lateral view (Figure 17H, Figure 20H)…………*subtilis* Knight

- Aedeagus basal process between shafts, in lateral view directed dorsally……………….……………………………………………8

8. Aedeagal shaft narrowed and prolonged beyond gonopore in posterior view; gonopore near midlength of shaft (Figure 17G, Figure 20G)……………………………*tenuis* sp. nov.

- Aedeagal shaft not narrowed and prolonged beyond gonopore in posterior view; gonopore near apex (Figure 17F, Figure 20F)……………………………………*knightiella* Virktamath & Murthy

9. Aedeagal shaft in lateral view with a projection in middle third………………………………………………………………………10

- Aedeagal shaft in lateral view without projection in middle third………………………………………………………………………15

10. Aedeagal shaft in lateral view with the process near middle shorter than width of shaft …………………………………………11

- Aedeagal shaft in lateral view with the process near middle longer than width of shaft (Figure 21D)………………………………*hamuleus* sp. nov.

11. Aedeagal shaft in lateral view with the process near middle spike-like………………………………………………………………12

- Aedeagal shaft in lateral view with the process near middle triangular or auricular…………………………………………………13

12. Aedeagal shaft in posterior view tapered before anterior hook (Figure 17I, Figure 21A)…………………………………………*diffractus* Dai, Fletcher & Zhang

- Aedeagal shaft in posterior view not tapered before anterior hook (Figure 18B, Figure 21C)………………………………………*ventralis* Cai & He

13. Aedeagal shaft in posterior view with process near middle auriform (Figure 18F)………………………………………………*fuscomaculatus* Li & Wang, 2004

- Aedeagal shaft in lateral view with process near middle triangular……………………………………………………………………14

14. Aedeagal shaft without apical processes in posterior view (Figure 19A)……………………………………………………………*viraktamathiellus* sp. nov.

- Aedeagal shaft with apical tooth at inner margin in posterior view (Figure 19B)……………………………………………………*triangulus* sp. nov.

15. Apex of aedeagal shaft broadly rounded or obliquely truncate………………………………………………………………………16

- Apex of aedeagal shaft filiform………………………………………………………………………………………………………………19

16. Aedeagal shaft with apical hood-like expansion anteriorly, gonopore elongate, extending approximately two thirds length of shaft (Figure 19E)………*bucephalus* Emeljanov

- Aedeagal shaft without apical hood-like expansion apically, gonopore short, subapical……………………………………………17

17. Aedeagal shaft with lateral margin straight; inner margin of the apex produced laterally into a process………………………18

- Aedeagal shaft with lateral margins concave near midlength, inner margin not produced into laterally directed process (Figure 19H)…………*sellatus* (Uhler)

18. Aedeagal shaft with apex approximately circular (Figure 18I)…………………………………………………………………………*paraphycitis* sp. nov.

- Aedeagal shaft with apexquadrate with two distinct angles (Figure 18H)………………………………………………………………*phycitis* (Distant)

19. Aedeagal shaft apex anteriorly hooked……………………………………………………………………………………………………20

- Aedeagal shaft apex anteriorly not hooked…………………………………………………………………………………………………23

20. Aedeagal shaft apex with lateral margins reflexed………………………………………………………………………………………21

- Aedeagal shaft apex with margins not reflexed……………………………………………………………………………………………22

21. Aedeagal shaft with double hook at apices (Figure 12G, Figure 18G)…………………………………………………………………*lii* sp. nov.

- Aedeagus shafts apex with outer margin hooked extension medially (Figure 18D)……………………………………………………*yuanmouensis* sp. nov.

22. Aedeagal shaft apex slender and elongate beyond gonopore (Figure 18A)……………………………………………………………*expansivus* Li

- Aedeagal shaft apex stouter beyond gonopore (Figure 18E)………………………………………………………………………………*hamatus* Kuoh

23. Aedeagal shaft with triangular lamellate expansion on anterior surface………………………………………………………………24

- Aedeagal shaft without such expansion………………………………………………………………………………………………………25

24. Aedeagal shaft with triangular lamellate expansion near midlength on anterior surface (Figure 22A)……………………………*concavus* Knight

- Aedeagal shaft with small triangular expansion subapically on anterior surface (Figure 22D)…………………………………………*fletcheri* sp. nov.

25. Aedeagal shaft with apex truncate…………………………………………………………………………………………………………*truncatus* Kuoh

- Aedeagal shaft apex not truncate………………………………………………………………………………………………………………26

26. Aedeagal shaft with short, thorn-like processes at apex (Figure 22B)…………………………………………………………………*dietrichi* sp. nov.

- Aedeagal shaft without processes at apex……………………………………………………………………………………………………27

27. Aedeagal shaft apex bifid (Figure 19G)……………………………………………………………………………………………………*dichotomous* Kuoh

- Aedeagal shaft apex entre not bifid (Figure 19I)……………………………………………………………………………………………*rectus* Kuoh

### 3.2. Species Description

#### 3.2.1. *Hishimonus prolongatus* Li & Wang, 2004

**Figure 1:**
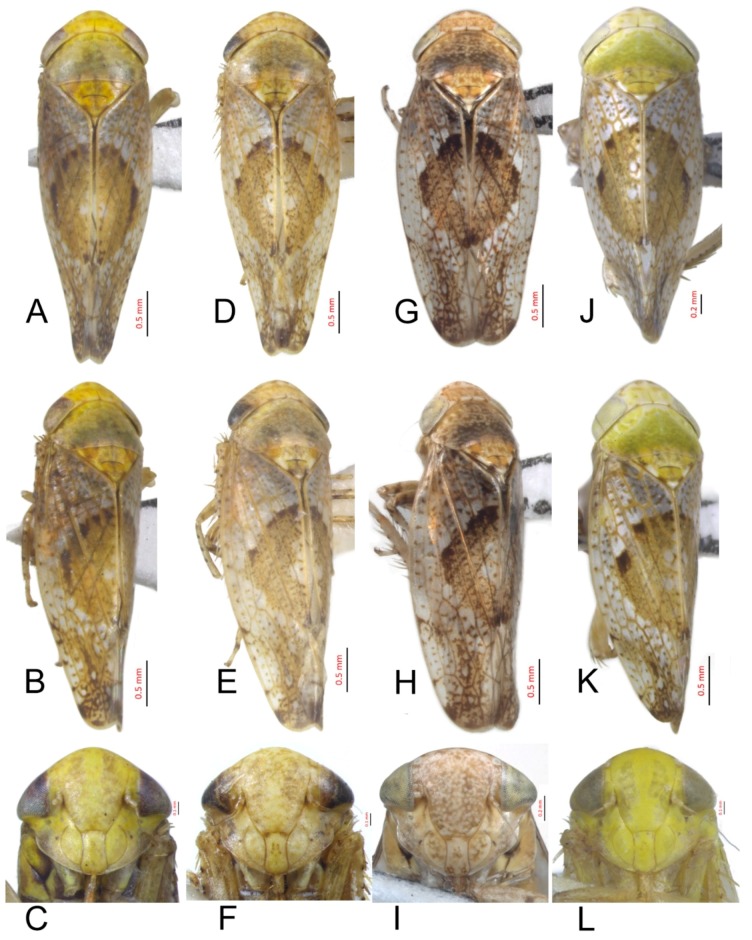
Species of *Hishimonus*. (**A**–**C**) *H. prolongatus*; (**D**–**F**) *H. spiniferous*; (**G**–**I**) *H. kuohi*; and (**J**–**L**) *H. tortuosus*. (**A**,**D**,**G**,**J**) Dorsal view; (**B**,**E**,**H**,**K**) latero-dorsal view; (**C**,**F**,**I**,**L**) Face.

Figure 1A–C, Figure 8A, Figure 11A, Figure 14A, Figure 17A and Figure 20A.

*Hishimonus prolongatus* Li & Wang, 2004: 487, Figures 1–6 [13]; Li, Dai & Xing, 2011: 95, Figures 5-84(1–6) [10].

**Description.** Length: ♂ 2.4–3.9 mm. Vertex pale greenish yellow, with two small darker spots apically and two darker markings anterior along shallow transverse depression. Face pale greenish yellow. Pronotum brownish yellow with indistinctly delimited fuscous spots. Scutellum brownish yellow with basal angle bright brown. Forewings silvery gray with densely dark brown mottling between veins, with brown, darker apically, with line of brown spots inside costal margin; brown median diamond patch distinct, well defined anteriorly by darker brown, with pale area medially.

**Male genitalia.** Pygofer in lateral view longer than its height, posterior margin rounded. Valve approximately twice as wide at base as long medially. Subgenital plate relatively long, broadly rounded at base, then lateral margins slightly curved and posteriorly abruptly rounded truncate to elongate posterior finger-like process. Style with preapical lobe well developed, apophysis slender and very long, about 2/5 as long as the total length of style. Connective with stem broad, slightly longer than arms. Aedeagus in posterior view V-shaped, with dorsal apodeme narrow, shafts cylindrical and straight, apex rounded with a moderate process at inner margin directed laterally; in lateral view with dorsal apodeme about half of shaft, evenly curved, shaft straight and slightly tapering to apex, with an apical long process directed laterally and basal processes about 1/2 as long as the length tapering to apex; gonopore apical.

**Female.** Unknown.

**Material examined.** 1♂, Sichuan, Emeishan, 15.vii.2011, Zhang Huining.

**Distribution.** China (Sichuan, Yunnan).

**Notes.** This species was described based on a male and a female specimen from Yunnan. Unfortunately, the illustrations given by Li and Wang [13] were misleading. They overlooked the preatrial processes, which are delicate, and unless carefully dissected, are likely to be damaged. Dr. Xing (personal communication) confirmed that the male genitalia illustrated here agreed with the male genitalia of the holotype. This species resembles *H. spiniferous* Kuoh but differs from the latter by the straight aedeagal shaft and long apical process.

#### 3.2.2. *Hishimonus spiniferous* Kuoh, 1976

Figure 1D–F, Figure 8B, Figure 11B, Figure 14B, Figure 17B, Figure 20B, Figure 23A and Figure 24A1–A3.

*Hishimonus spiniferous* Kuoh, 1976: 435, Figure 7a–f [11]; Li, 1987: 305, Figure 154 [41]; Li & Wang, 1991: 201 [42]; Li & Wang, 2004: 486 [13]; Xing & Li, 2010: 134 [43]; Li, Dai & Xing, 2011: 98, Figures 5–88(1–5) [10]; and Xing, Dai & Li, 2012: 168 [44].

**Description.** Length: ♂ 3.6–4.3 mm, ♀ 4.1–5.0 mm. Head and thorax yellowish ivory. Vertex with darker spots apically and transverse marking anterior along shallow transverse depression. Face with indistinct darker spots. Pronotum brownish yellow with indistinctly fuscous spots. Scutellum brownish yellow with dark brown basal triangles and apical half, apex and one spot on each lateral margin ivory. Forewings silvery gray with dark brown mottling between veins, becoming darker and more dense apically; brown median diamond patch distinct, well-defined anteriorly by dark brown with pale area medially.

**Male genitalia.** Pygofer in lateral view longer than its height, posterior margin rounded. Valve approximately twice as wide at base as long medially. Subgenital plate broadly triangular, rounded laterally at base, then rounded to posterior finger-like process. Style with preapical lobe well-developed and forming right angle, apophysis very long and straight, curved slightly medially along length to be almost parallel with opposing style apophysis. Connective with stem broad, slightly longer than arms. Aedeagus in posterior view with dorsal apodeme slender, shafts straight and cylindrical, gradually narrowed to acutely rounded apex, with a pair of basal process; in lateral view with dorsal apodeme about 0.66 as long as shaft, moderately curved, shaft elongate, curved dorsally in midlength, with a reflexed short apical process and ventral processes about 1/2 as long as the length at base; gonopore subapical on posterior surface.

**Female.** Sternite VII nearly twice as wide as long, posterior margin broad V-shaped with pair of short median triangular projections separated by V-shaped emargination. Second valvulae with dorsal margin broadly arcuate, dorsal teeth moderately prominent and near middle than at apex.

**Material examined.** 10♂♂4♀♀, Gansu, Chengxian, 25.vii.2002, Wei Cong & Shang Suqin; 5♀♀, Gansu, Kangxian, 1.vii.2002, Wei Cong & Yang Zhaofu; 9♂♂, Guizhou, Bijie, 9.vii.2018, Liang Zonglei; 16♂♂2♀♀, Guizhou, Tongren, 26.vii.2018, Liang Zonglei; 1♂, Guizhou, Fanjingshan, 1.viii.2001, Sun Qiang; 1♂, Hubei, Wufenghouhe, 13.vii.2006, Lv Lin; 1♂, Hubei, Shennongjia, 19.vii.2003, Yang Yongjun; 2♀♀, Hubei, Jiugongshan, 6.vii.2001, Huang Min & Che Yanli; 2♀♀, Hubei, Wudangshan, 20.vii.2001, Huang Min; 1♂2♀♀, Hubei, Fangxian, 21.vii.2001, Huang Min & Che Yanli; 1♂1♀, Shaanxi, Nanzheng, 23.vii.2004, Lv Lin & Duan Yani; 2♂♂6♀♀, Shaanxi, Lueyang, 19.viii.2002, Wei Cong & Shang Suqin; 2♀♀, Shaanxi, Yangxian, 22.viii.2002, Wei Cong & Shang Suqin; 1♂2♀♀, Shaanxi, Liuba, 19.viii.1995; 1♂20♀♀, Shaanxi, Fengxian, 14.viii.1995, Zhang Wenshu & Ren Liyun; 5♂♂, Sichuan, Emeishan, 16.vii.2011, Zhang Huining; 1♂1♀, Sichuan, Batang, 11.vii.2007, Sun Qiang; 1♂, Sichuan, Laohegou, 22.vii.2014, Yang Liyuan; and 1♂, Yunnan, Dali, 15.viii.2018, Liang Zonglei.

**Distribution.** China (Gansu, Guizhou, Hainan, Hubei, Shaanxi, Sichuan, and Yunnan).

**Remarks.***H. spiniferous* was described by Kuoh [11] based on two male and four female specimens from Yunnan. It was similar to *H. prolongatus* Li & Wang, but differed from the latter in the short apical process of aedeagal shaft, which was curved dorsally at midlength and in the round anterior margin of the subgenital plates at the base of the apical process.

#### 3.2.3. *Hishimonus kuohi* Du & Dai, sp. nov.

Figure 1G–I, Figure 8C, Figure 11C, Figure 14C, Figure 17C, Figure 20C, Figure 23B and Figure 24B1–B3.

**Description.** Length: ♂ 3.4–4.0 mm, ♀ 3.7 mm. Head and pronotum yellowish ivory with brown speckling. Vertex with two spots at apex and darker markings along shallow transverse depression. Face yellowish ivory, scattered many brown mottling. Scutellum pale brownish yellow, with basal triangles and apical half dark brownish yellow, apex and one spot on each lateral margin ivory. Forewings silvery white with dark brown mottling between veins, becoming darker and more dense apically; brown median diamond patch distinct, well-defined by dark brown at anterior margin and posterior corner, with pale area medially.

**Male genitalia.** Pygofer in lateral view slightly longer than height, posterior margin bluntly rounded. Valve approximately twice as wide as long medially. Subgenital plate extended well beyond apex of pygofer, rounded externally to posterior finger-like process. Style with preapical lobe moderately developed and forming right angle, apophysis very long. Connective with stem broad, equal to arms. Aedeagus in posterior view with dorsal apodeme broad at base and constricted narrowed to apex, shafts divergent in basal half and slightly curved mesally slightly beyond midlength; in lateral view with dorsal apodeme 2/3 as long as aedeagal shaft; shaft moderately long, cylindrical, apex curved dorsally, with ventral processes about 2/3 as long as the length of shaft at base; gonopore subapical on posterior surface.

**Female.** Sternite VII twice as wide as long, posterior margin broad V-shaped with a black, M-shaped medial lobe separated by relatively deep lateral concavity. Second valvulae with dorsal margin more or less arcuate, and dorsal teeth relatively small.

**Material examined.** Holotype: ♂, Yunnan, Yuanmou, 27.vi.2005, Li Qiao; Paratypes: 2♂♂1♀, Guizhou, Guanling, 8.viii.2016, Wen Chao; 2♂♂1♀, Yunnan, Hutiaoxia, 4.viii.2015, Du Yimin (NWAFU).

**Etymology:** The species is named after Prof. Chunglin Kuoh of Anhui Agricultural University in recognition of their contributions to leafhopper taxonomy.

**Distribution.** China (Guizhou, Yunnan).

**Remarks.** This species is similar to *H. spiniferous* Kuoh, but can be distinguished by the straight aedeagal shafts.

#### 3.2.4. *Hishimonus tortuosus* Kuoh, 1976

Figure 1J–L, Figure 8D, Figure 11D, Figure 14D, Figure 17D, Figure 20D, Figure 23C and Figure 24C1–C3.

*Hishimonus tortuosus* Kuoh, 1976: 434, Figure 6a–f [11]; Li & Wang, 2004: 487 [13].

**Description.** Length: ♂ 3.5–3.7 mm, ♀ 3.9–4.2 mm. Vertex, face and pronotum pale greenish yellow, with indistinctly fuscous spots. Scutellum pale greenish yellow marked with pale brown, with apex and one spot on each lateral margin ivory. Forewings silvery with little dark brown mottling between veins, becoming darker and more dense apically; brown median diamond patch well-defined by dark brown at anterior margin, with pale area medially.

**Male genitalia.** Pygofer in lateral view longer than height, posterior margin acutely rounded. Valve approximately 2.5 times as wide as long medially. Subgenital plate moderately long and extended well beyond apex of pygofer, relatively broad, rounded externally to posterior finger-like process. Style with preapical lobe developed and forming right angle, apophysis short, finger-like. Connective with stem broad, shorter than arms. Aedeagus in posterior view shaft broad divergent basally, gradually narrowed and curved laterally near apex, and then tapered distally to pointed apex, basal processes straight, longer than shafts, tapering apically; in lateral view with dorsal apodeme nearly 1/2 as long as shaft, shaft short and robust, gradually narrowed near apex, abruptly tapering at apex, basal processes curved dorsally; gonopore subapical.

**Female.** Sternite VII less than twice as wide as long, tapered, posterior margin broad shallow concave, with pair of small median lobes separated by relatively shallow concavity. Second valvulae with dorsal margin biarcuate, teeth numerous, short, blunt.

**Material examined.** 1♂1♀, Guangdong, Dinghushan, 14.vii.2017, Du Lan; 1♂, Hainan, Xinglong, 28.iv.1983, Zhang Yalin; and 1♂, Hainan, Jianfengling, 13.iv.2018, Du Lan.

**Distribution.** China (Guangdong, Hainan).

**Remarks.***H. tortuosus* was described by Kuoh [11] on the basis of material from Hainan. This species can easily be recognized among the species from China by the basal process of the aedeagal shaft, which is straight and longer than the shaft. The Indian species *H. longistylus* Viraktamath & Murthy also has the basal processes longer than the shaft, but the processes are sinuate.

#### 3.2.5. *Hishimonus aberrans* Knight, 1970

Figure 2A–C, Figure 8E, Figure 11E, Figure 14E, Figure 17E, Figure 20E, Figure 23D and Figure 24D1–D3.

Hishimonus aberrans Knight, 1970: 137–138, Figures 68–71 [9]; Kuoh, 1976: 8, Figure 10 [11]; Li & Wang, 2004: 487 [13]; Li, Dai & Xing, 2011: 89, Figures 5–76(1–5) [10]; Dai, Fletcher & Zhang, 2013: 308, Figures 1C, 1F, 5A–F [7]; and Viraktamath & Murthy, 2014: 106, Figures 1–2, 42–53 [8].

**Description.** Length: ♂ 3.4–4.0 mm, ♀ 3.5–3.8 mm. Head and thorax pale testaceous with brown speckling. Vertex with darker markings anterior and posterior along shallow transverse depression. Pronotum scattered brown mottling denser posteriorly. Scutellum with basal triangles and apical half brown, with apex and one spot on each lateral margin ivory. Forewings brownish with darker mottling denser apically and with series of dark spots inside costal margin; brown median diamond patch indistinct, more even texture than rest of forewing, with pale spots medially.

**Male genitalia.** Pygofer in lateral view longer than its height, posterior margin rounded. Valve approximately twice as wide at base as long medially. Subgenital plates broadly rounded at base, lateral margins lightly curved and abruptly roundly truncate to narrow posterior finger-like process. Style with preapical lobe undeveloped, apophysis long, tapering. Connective with stem broad, slightly shorter than arms. Aedeagus with shafts moderately divergent in posterior view, curved dorsally, almost parallel towards apex, with narrow elongate basal process from posterior edge of each shaft extending to about half length of shafts. In lateral view, shafts more or less straight to almost midlength then curved anteriorly, with apical process hooked anteriorly, basal process slightly tapering from base to apex; gonopore subapical on posterior surface.

**Female.** Female seventh sternite 2.5 times as wide at base as long medially, posterior margin broad concave, with a V-shaped median notch, posterolateral angles acutely rounded. First valvulae about 2/3 as wide as long. Second valvulae with dorsal margin nearly straight through most of length, teeth not prominent, rounded and somewhat irregularly spaced.

**Material examined.** 4♂♂1♀, Hainan, Yinggeling, 5.v.2018, Du Lan; 2♂♂1♀, Yunnan, Dali, 13.v.2013, Xue Qingquan.

**Distribution.** China (Hainan, Taiwan, Yunnan), India, Thailand.

**Remarks.** This species was described by Knight [9] based on a single male specimen from Taiwan. It shows affinity with *H. diffractus* Dai, Fletcher & Zhang in which the basal processes are united with the shaft of the aedeagus on each side rather than being separate from the shaft throughout their length as in this species. Both species have the subgenital plates abruptly narrowed apically before the apical finger-like process, a feature they also share with *H. gillespiei* Dai, Fletcher & Zhang.

#### 3.2.6. *Hishimonus knightiellus* Viraktamath & Murthy, 2014

Figure 2D–F, Figure 8F, Figure 11F, Figure 14F, Figure 17F, Figure 20F, Figure 23E and Figure 24E1–E3.

Hishimonus apricus, Knight, 1970: 135, Figures 58–59 [9]. Preoccupied by *Eutettix apricus* Melichar, 1903: 190–192 [19].

*Hishimonus knightiella* Viraktamath & Murthy, 2014: 107 [8].

**Description.** Length: ♂ 3.3–3.7 mm, ♀ 3.5–3.9 mm. Vertex pale greenish yellow, with two light brown transverse mottling near apex and several on posterior margin. Pronotum greenish yellow on anterior half while the posterior half somewhat dark, with several spots on either side of anterior margin. Scutellum greenish yellow, with two spots on anterior side of transverse impressed line and two on lateral margins on each side, light brown, apex and one spot on each side of anterior margin ivory white. Forewings silvery white with pale brown mottling between veins, becoming darker and more dense apically; brown median diamond patch distinct, well-defined by dark brown at anterior margin and posterior corner, with median section anterior to posterior patch translucent whitish.

**Male genitalia.** Pygofer approximately as long as height in lateral view, posterior margin slightly angulate. Valve twice as wide as long medially. Subgenital plates broadly rounded at base, and then margins lightly curved and tapering to narrow posterior finger-like process. Style with developed preapical lobe, apophysis short and slightly tapering to apex. Connective with stem especially short, approximately 1/2 as long as to arms. Aedeagus with shafts moderately diverged, almost parallel towards acute apex in posterior view, a pair of basal processes insert between base of the two shafts, arriving at about half length of shafts; in lateral view, shafts slightly curved and gradually tapering to acute apex; gonopores subapical on posterior surface.

**Female.** Sternite VII nearly 2.5 times as wide as long, posterior margin almost straight, medial portion slightly wavy and concave. Second valvulae with dorsal teeth moderately prominent.

**Material examined.** 14♂♂7♀♀, Hainan, Yinggeling, 5.v.2018, Du Lan.

**Distribution.** China (Hainan, Taiwan), Borneo, Malaysia, Singapore, and Sri Lanka.

**Remarks.** This species was described by Knight [9] who ignored *Eutettix apricus* Melichar, 1903 and named it as *Hishimonus apricus*, a secondary homonym. The current scientific name *Hishimonus knightiella* was proposed as a nomen novum by Viraktamath & Murthy in 2014 [8]. The species name is here emended to *Hishimonus knightiellus* to agree with the genus name in gender. It can be identified by the widely placed basal processes that are almost equidistant from the base of the dorsal apodeme and the base of the shaft as well as by the extended outer edge of the shaft apex. It is recorded here for the first time from China.

#### 3.2.7. *Hishimonus tenuis* Du & Dai, sp. nov.

Figure 2G–I, Figure 8G, Figure 11G, Figure 14G, Figure 17G, Figure 20G, Figure 23F and Figure 24F1–F3.

**Description.** Length: ♂ 3.5–4.0 mm, ♀ 3.5–3.8 mm. Vertex, pronotum, and scutellum ivory yellow. Vertex with anterior marginal and submarginal light brown mottling on either side of coronal sulcus, and several spots on posterior margin. Pronotum with irregular pale spots on anterior margin and slightly dark brown mottling on pronotum posteriorly. Scutellum with basal triangles and apical half pale brownish yellow, apex and one spot at each side of lateral margin ivory white. Forewings silvery white with brown mottling between veins, becoming darker and more dense apically; brown median diamond patch distinct, well-defined by dark brown at anterior margin, with median section anterior to posterior translucent whitish.

**Male genitalia.** Pygofer longer than height in lateral view, dorsal margin strongly sinuate in lateral aspect. Valve broadly triangular with caudal margin produced acutely, approximately twice as wide as medially long. Subgenital plates broadly rounded at base, margins lightly narrowing to elongate posterior finger-like process. Style with preapical lobe developed, apophysis moderately long and slender, tapering to apex. Connective with stem broad, about 1/2 as long as arms. Aedeagus in posterior view with shafts straight and moderately divergent, narrowing abruptly from midlength, tapering towards apex and slightly curved medially, one pair of stout processes inserted posterad of dorsal apodeme and between base of shafts; in lateral view, shafts robust and abruptly tapering near apex and slightly curved ventrally; gonopore subapical on posterior surface.

**Female.** Female seventh sternite 2.5 times as wide at base as long medially, posterior margin truncate. First valvulae about 0.66 as wide as long. Second valvulae with dorsal teeth prominent.

**Material examined.** Holotype: ♂, Yunnan, Yexianggu, 26.vii.2011, Zhang Huining; Paratypes: 3♂♂2♀♀, data same as holotype; 43♂♂21♀♀, Yunnan, Yexianggu, 26.vii.2017, Liang Zonglei; 9♂♂5♀♀, Yunnan, Yexianggu, 13.v.2011; 2♂♂, Yunnan, Jinghong, 30.viii.2010, Han Juan; 3♂♂8♀♀, Yunnan, Mengla, 18.v.2011; 1♂1♀, Yunnan, Mengla, 5.v.1974, Zhou Yao & Yuan Feng; 1♀, Yunnan, Mengla, 3.v.1991, Wang Yinglun & Tian Rungang; 1♀, Yunnan, Mengla, 9.v.1991, Liu Guangchun & Cai Wanzhi; 1♂1♀, Yunnan, Menglun, 19.v.1991, Wang Yinglun & Tian Rungang; 1♂, Yunnan, Menglun, 21.iv.1982, Zhou Jingruo & Wang Sumei; and 1♀, Yunnan, MengYang, 9.vi.1991, Wang Yinglun & Tian Rungang (NWAFU).

**Distribution.** China (Yunnan).

**Etymology.** The species name refers to short aedeagal shaft.

**Remarks.***H. tenuis* is similar to *H. subtilis* Knight, but it can be distinguished by the stouter basal processes (in posterior view), which are not sinuate in lateral view (in *H. stubtilis*, the basal processes are thinner in posterior view and sinuate in lateral view).

#### 3.2.8. *Hishimonus subtilis* Knight, 1970

Figure 2J–L, Figure 8H, Figure 11H, Figure 14H, Figure 17H, Figure 20H, Figure 23G and Figure 24G1–G3.

Hishimonus subtilis Knight, 1970: 135, Figures 51–52 [9]; Dai, Fletcher & Zhang, 2013: 305, Figures 1B, 1E, 4A–F [7].

**Description.** Length: ♂ 2.9 mm, ♀ 3.5–4.0 mm. Head and thorax pale greenish yellow scattered brown mottling. Forewing silvery white, with scattered brown mottling between veins, becoming slightly darker and dense apically; brown median diamond patch distinct, surrounded by dark brown at anterior margin and posterior corner, with median section anterior to posterior patch translucent whitish.

**Male genitalia.** Pygofer longer than height in lateral view, dorsal margin strongly sinuate in lateral aspect. Valve approximately twice as wide at base as long medially. Subgenital plates broadly rounded at base then rounded to elongate posterior process. Style with preapical lobe moderately developed, apophysis tapering and slightly curved laterally. Connective with stem broad, one-half as long as arms. Aedeagus with shafts moderately divergent in posterior view, narrowing abruptly from basal 1/3, tapering towards apex and slightly curved medially, with median pair of short straight processes anterior to base of shafts. In lateral view, shafts curving dorsally, broad to level of gonopore then extended from anterior side of gonopore as elongate curved narrow apical process curved slightly anteriorly; gonopore on posterior surface at midlength.

**Female.** Sternite VII nearly twice as wide as long, posterior margin almost straight, slightly sinuate medially. First valvulae with apex sagittal. Second valvulae with dorsal teeth prominent.

**Material examined.** 1♂10♀♀, Hainan, Jianfeng, v.2011, Jin Li.

**Distribution.** China (Hainan), Malaysia, Singapore, Thailand.

**Remarks.** This species was described by Knight [9] based on a single male specimen from Singapore. It is the only species recorded in China with a pair of basal processes anterior to the bases of the aedeagal shafts, regarded by Knight [9] as a derived feature. The apical aedeagal process may represent the apical portion of a long basal process that has become fused with the aedeagal shaft throughout its length and now appears as an apical extension. It is recorded here for the first time from China.

#### 3.2.9. *Hishimonus diffractus* Dai, Fletcher & Zhang, 2013

**Figure 3:**
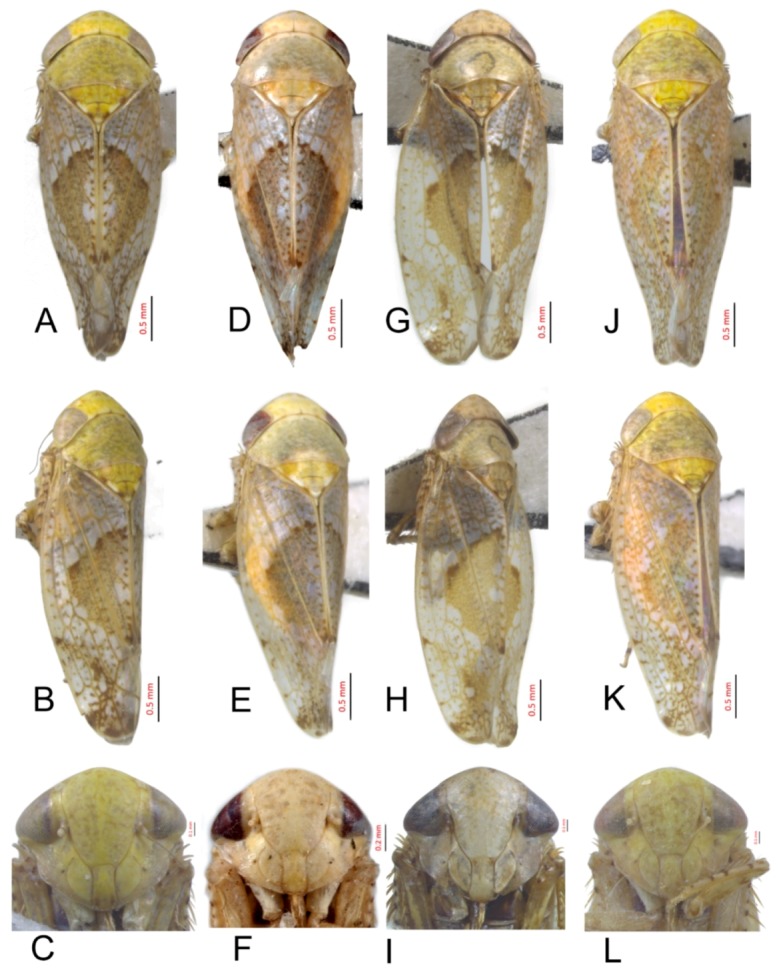
Species of *Hishimonus*. (**A**–**C**) *H. diffractus*; (**D**–**F**) *H. expansivus*; (**G**–**I**) *H. ventralis*; and (**J**–**L**) *H. hamuleus*. (**A**,**D**,**G**,**J**) Dorsal view; (**B**,**E**,**H**,**K**) latero-dorsal view; and (**C**,**F**,**I**,**L**) Face.

Figure 3A–C, Figure 8I, Figure 11I, Figure 14I, Figure 17I and Figure 21A.

Hishimonus diffractus Dai, Fletcher & Zhang, 2013: 311, Figures 2C, 2F, 8A–E [7]; Fletcher & Dai, 2013: 423, Figures 1B, 4C–E [6].

**Description.** Length: ♂ 3.8 mm. Vertex, pronotum, and scutellum greenish yellow marked with pale brown patches. Scutellum with lateral and apical areas ivory white, marked with two brown spots near base and another before apex. Forewings silvery white with brown mottling between veins, becoming darker and denser apically; brown median diamond patch distinct, well defined by dark brown at anterior margin and posterior corner, with median section anterior to posterior patch translucent whitish.

**Male genitalia.** Pygofer in lateral view longer than height, posterior margin rounded. Valve approximately twice as wide at base as long medially. Subgenital plates broad triangular, broadly rounded at base then rounded apically to posterior finger-like process from median corner. Style with apophysis long and thick, apically rounded. Connective with stem broad, about as long as lateral arms. Aedeagal shafts, in posterior view, more or less straight, divergent of even width throughout and bearing short, sharp thorn-like process on inner margin at about one-half to two-thirds length. In lateral view, shafts evenly lightly curved from base with apical process much narrower and strongly reflexed anteriorly; gonopore subapical on posterior surface.

**Female.** Unknown.

**Material examined.** 2♂♂ (Holotype), China: Jiangxi, Suichuan, 13.viii.2004, Wei Cong & Yang Meixia.

**Distribution.** China (Jiangxi), Australia, Thailand.

**Remarks.** This species has closest affinity with *H. aberrans* Knight from China and Thailand, which also has the subgenital plates abruptly narrowed before the apical process. However, the basal processes, which are separate from the shaft for almost their entire length in *H. aberrans,* are entirely fused with the shafts in *H. diffractus* except for the apical portion, which remains as a short, spine-like process at about two-thirds of the length of the shaft.

#### 3.2.10. *Hishimonus expansivus* Li, 1988

Figure 3D–F, Figure 9A, Figure 12A, Figure 15A, Figure 18A, Figure 21B, Figure 23H and Figure 24H1–H3.

Hishimonus expansivus Li, 1988: 51, Figure 1 [12]; Li & Wang, 1991: 201, Figures VII-3, 104 [42]; and Li, Dai & Xing, 2011: 92, Figures 5–80(1–5) [10].

*Hishimonus lamellatus* Cai & Kuoh, 1995: 217, Figures 1–8 [45]; Li & Wang, 2004: 487 [13]. **syn. nov.**

**Description.** Length: ♂ 3.7–4.5 mm, ♀ 3.9–5.0 mm. Vertex, pronotum and scutellum stramineous. Scutellum with basal triangles light brown, apex and one spot at each side of lateral margin ivory. Forewings silvery white with brown mottling between veins, becoming darker and denser apically; brown median diamond patch surrounded by dark brown margins, with median section anterior to posterior translucent whitish.

**Male genitalia.** Pygofer in lateral view slight longer than height, posterior margin rounded. Valve triangular with caudal margin produced acutely, approximately twice as wide as medially long. Subgenital plates broadly rounded at base, lateral margins lightly roundly curved to elongate posterior finger-like process. Style with preapical lobe undeveloped, apophysis long, tapering to apex. Connective with stem slightly shorter than arms. Aedeagus in posterior view with dorsal apodeme narrow, shaft constricted at base, widen triangularly to both sides near midlength, then narrowing towards rounded apex with slight medial notch; in lateral view, shafts slightly curved dorsally, with apical process much narrower and strongly reflexed anteriorly; gonopore subapical on posterior surface.

**Female.** Sternite VII nearly twice as wide as long, posterior margin slightly concaved, with two median denticles separated by tiny notch. Second valvulae with dorsal margin slightly arcuate, teeth prominent.

**Material examined.** 3♂♂3♀♀, Shaanxi, Yangling, 23.vi.1985, Zhang Yalin; 6♂♂, Shanxi, Jincheng, 18.vii.2012, Yang Liyuan.

**Distribution.** China (Hebei, Shaanxi, Shanxi).

**Remarks.***H. expansivus* was described by Li [12] based on a specimen collected in Guizhou and can be identified by the aedeagus with shafts expanded at the midlength and tapering at the apex. *H. lamellatus* was described by Cai, Cui & Kuoh in 1995 [45] based on a specimen from Hebei. Based on the descriptions and illustrations provided by Li [12] and Cai, Cui & Kuoh [45], we proposed the synonymy of these two taxa.

#### 3.2.11. *Hishimonus ventralis* Cai & He, 2001

Figure 3G–I, Figure 9B, Figure 12B, Figure 15B, Figure 18B and Figure 21C.

Hishimonus ventralis Cai & He in Cai, He & Gu, 2001: 207, Figures 97–103 [46]; Li & Wang, 2004: 487 [13].

**Description.** Length: ♂ 3.9–4.4 mm, ♀ 4.1 mm. Vertex, pronotum and scutellum brownish yellow marked with pale brown patches; scutellum with basal angle fawn. Forewings silvery white with brown mottling between veins, becoming darker and dense apically; brown median diamond patch distinct, surrounded by dark brown at anterior margin.

**Male genitalia.** Pygofer in lateral view longer than height, posterior margin rounded. Subgenital plate extended well beyond apex of pygofer, broadly rounded at base, and then margins lightly curved to elongate posterior finger-like process. Style with preapical lobe developed, apophysis very long, equal to one half of style, slightly tapering to apex. Connective with stem slightly shorter than arms. Aedeagus in posterior view with shafts moderately diverged, with a short process directed ventrally at the middle of shafts and an apical process curved dorsally; in later view, shafts curved dorsally and slight narrowed to rounded apex, with apical process much narrower and strongly reflexed anteriorly. Gonopores subapical on posterior surface.

**Female.** Unknown.

**Material examined.** 1♂, Zhejiang, Tianmushan, 24.viii.2000, Dai Wu; 2♂♂, Zhejiang, Tianmushan, 26.vi.2018, Hu Yulin.

**Distribution.** China (Zhejiang).

**Remarks.***H. ventralis* was described by Cai & He [46] based on a specimen from Zhejiang. It can be identified by the bent, hook-like processes as well as the spine-like process on the medial lateral margin of each aedeagal shaft.

#### 3.2.12. *Hishimonus hamuleus* Du & Dai, sp. nov.

Figure 3J–L, Figure 9C, Figure 12C, Figure 15C, Figure 18C, Figure 21D, Figure 23I and Figure 25A1–A3.

**Description.** Length: ♂ 3.9–4.0 mm, ♀ 4.3 mm. Vertex greenish yellow with indistinctly fuscous spots. Face with indistinctly fuscous spots. Pronotum greenish yellow marking fuscous patches posteriorly. Scutellum greenish yellow marked with brown spots on lateral margin, with apex and one spot on each lateral margin ivory. Forewings silvery gray with dense, pale brown mottling between veins, becoming darker and more dense apically; brown median diamond patch indistinct.

**Male genitalia.** Pygofer longer than height in lateral view, caudal margin slightly angulate. Valve likes regularly triangular, lateral margin narrowing apically. Subgenital plates broadly rounded at base, and then margins gradually convex to posterior finger-like process. Style with preapical lobe developed, apophysis long and slender, as long as 2/5 length of style, tapering to apex. Connective with stem broad, approximately 0.75 in length to arms. Aedeagus with shafts moderately diverged from the base, broad extending apically with two pairs of hook-like processes in posterior view; the outer margin process long and narrow at 2/3 length of shaft, pointing towards anterior; the apical process short and curved, reflexing towards anterior; in later view, shafts straight, slightly narrowed to apex, with a blade expansion at subapex and a hook-like apical process strongly reflexed anteriorly; gonopores subapical on posterior surface.

**Female.** Sternite VII less than twice as wide as long, posterior margin almost straight, with two median denticles separated by a tiny notch. Second valvulae with dorsal teeth prominent.

**Material examined.** Holotype: ♂, Guangxi, Dayaoshan, 10.ix.2000, Liu Zhenjiang; Paratypes: 12♂♂1♀, data same as holotype (NWAFU).

**Distribution.** China (Guangxi).

**Etymology.** The species name refers to the aedeagal shafts with two pairs of hook-like processes in posterior view**.**

**Remarks.** This species can be identified by the distinct hamulus at 2/3 length of each shaft as well as the reflexed process in lateral view. It is similar to *H. compactus* Knight, but the later has the shaft rounded at the apex and the process extending to near the base of the aedeagus.

#### 3.2.13. *Hishimonus yuanmouensis* Du & Dai, sp.nov.

**Figure 4:**
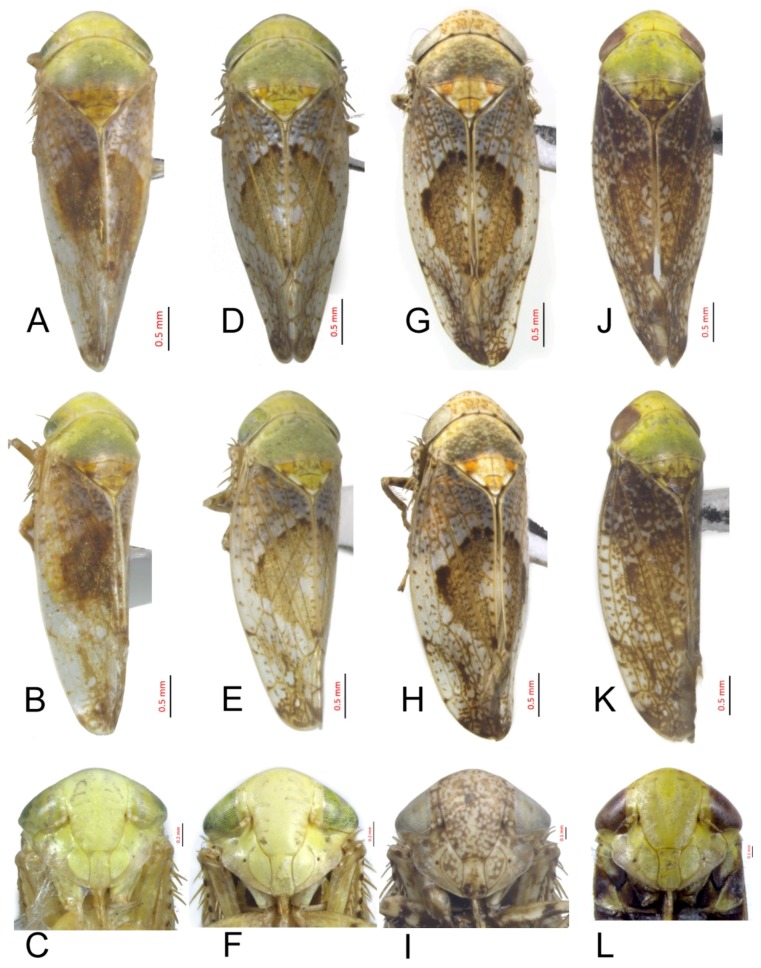
Species of *Hishimonus*. (**A**–**C**) *H. yuanmouensis*; (**D**–**F**) *H. hamatus*; (**G**–**I**) *H. fuscomaculatus*; and (**J**–**L**) *H. lii*. (**A**,**D**,**G**,**J**) Dorsal view; (**B**,**E**,**H**,**K**) latero-dorsal view; and (**C**,**F**,**I**,**L**) Face.

**Figure 8:**
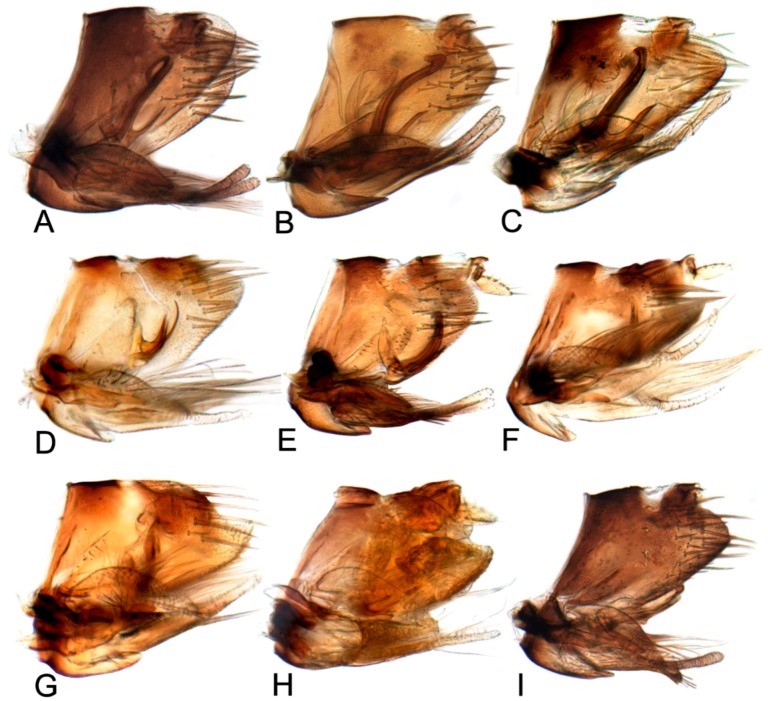
Male pygofer, lateral view: (**A**) *H. prolongatus*; (**B**) *H. spiniferous*; (**C**) *H. kuohi*; (**D**) *H. tortuosus*; (**E**) *H. aberrans*; (**F**) *H. knightiellus*; (**G**) *H. tenuis*; (**H**) *H. subtilis*; and (**I**) *H. diffractus*.

**Figure 9:**
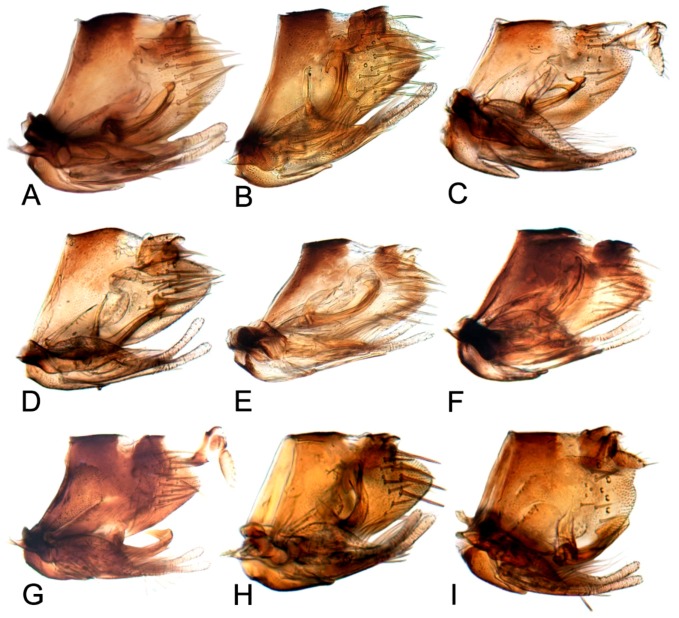
Male pygofer, lateral view: (**A**) *H. expansivus*; (**B**) *H. ventralis*; (**C**) *H. hamuleus*; (**D**) *H. yuanmouensis*; (**E**) *H. hamatus*; (**F**) *H. fuscomaculatus*; (**G**) *H. lii*; (**H**) *H. phycitis*; and (**I**) *H. paraphycitis*.

**Figure 11:**
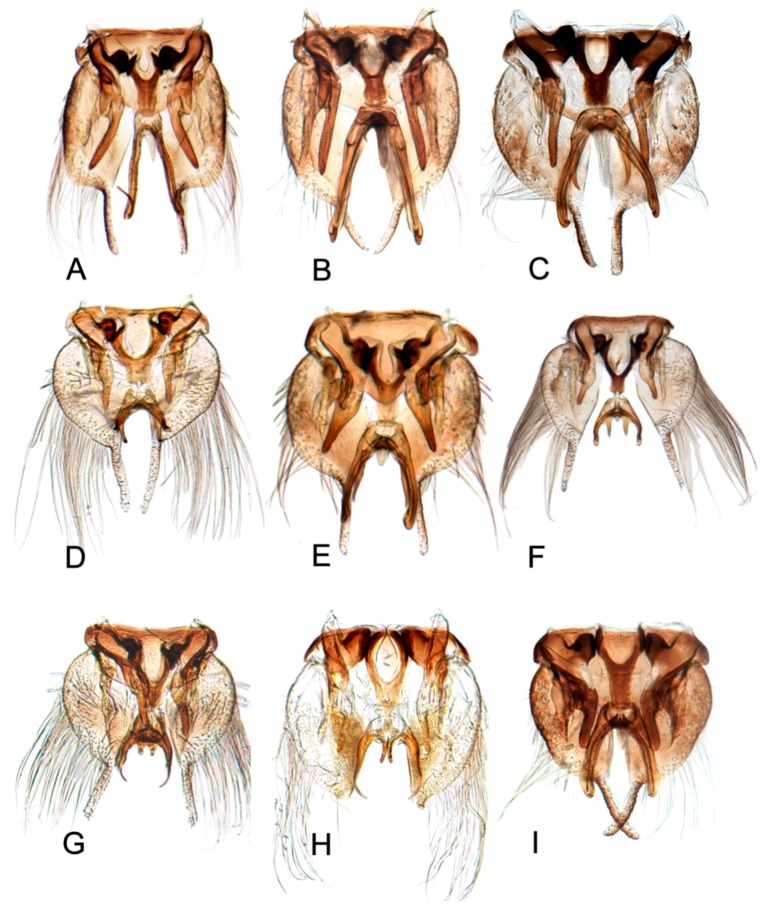
Subgenital plate, valve, style, connective, and aedeagus, dorsal view. (**A**) *H. prolongatus*; (**B**) *H. spiniferous*; (**C**) *H. kuohi*; (**D**) *H. tortuosus*; (**E**) *H. aberrans*; (**F**) *H. knightiellus*; (**G**) *H. tenuis*; (**H**) *H. subtilis*; and (**I**) *H. diffractus*.

**Figure 12:**
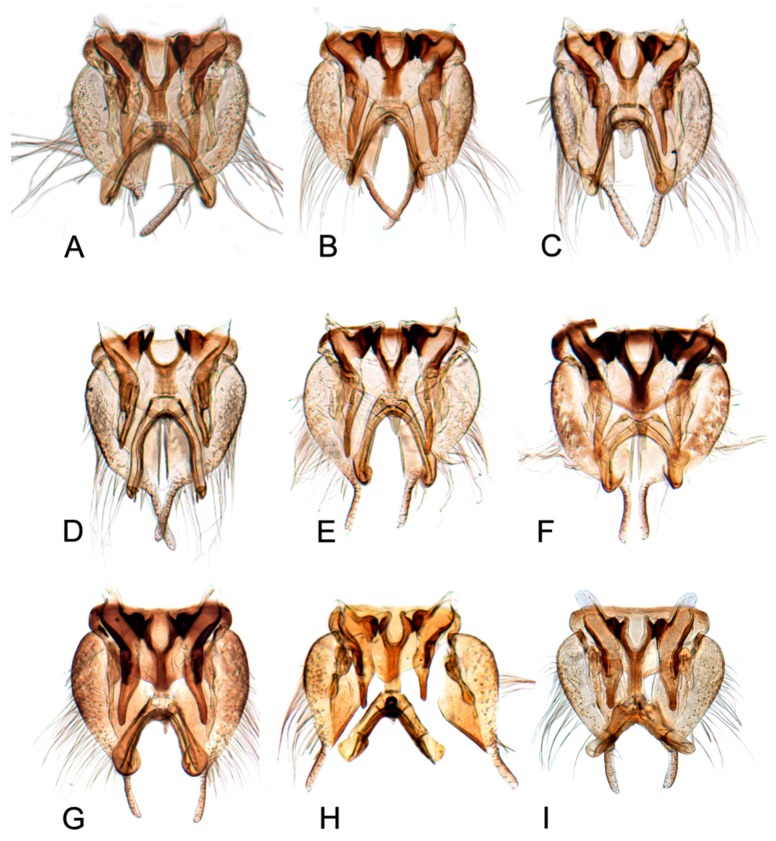
Subgenital plate, valve, style, connective, and aedeagus, dorsal view. (**A**) *H. expansivus*; (**B**) *H. ventralis*; (**C**) *H. hamuleus*; (**D**) *H. yuanmouensis*; (**E**) *H. hamatus*; (**F**) *H. fuscomaculatus*; (**G**) *H. lii*; (**H**) *H. phycitis*; and (**I**) *H. paraphycitis*.

**Figure 14:**
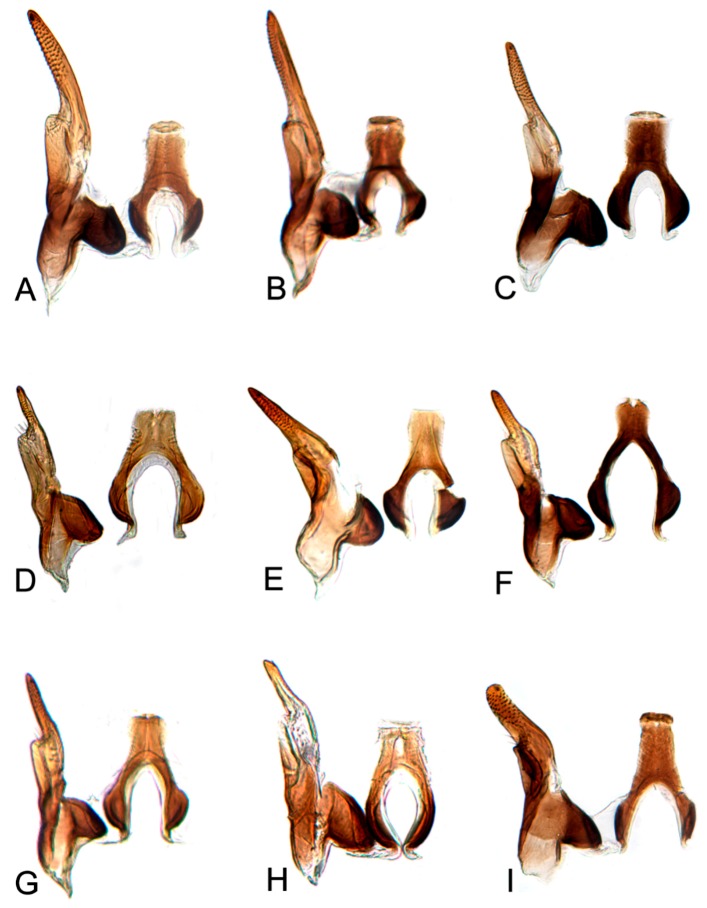
Style and connective, ventral view. (**A**) *H. prolongatus*; (**B**) *H. spiniferous*; (**C**) *H. kuohi*; (**D**) *H. tortuosus*; (**E**) *H. aberrans*; (**F**) *H. knightiellus*; (**G**) *H. tenuis*; (**H**) *H. subtilis*; and (**I**) *H. diffractus*.

**Figure 15:**
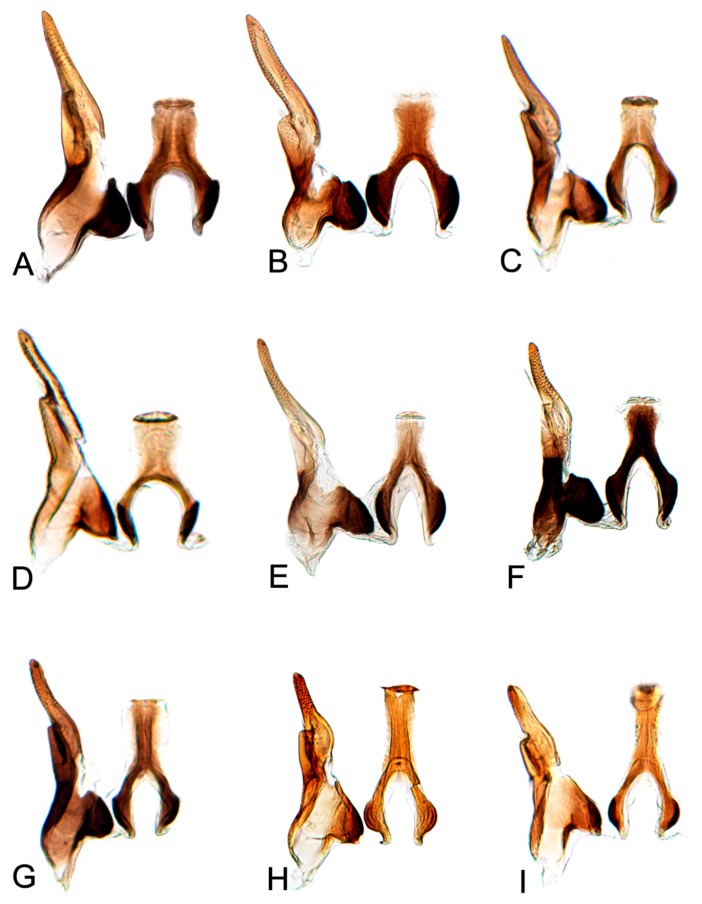
Style and connective, ventral view. (**A**) *H. expansivus*; (**B**) *H. ventralis*; (**C**) *H. hamuleus*; (**D**) *H. yuanmouensis*; (**E**) *H. hamatus*; (**F**) *H. fuscomaculatus*; (**G**) *H. lii*; (**H**) *H. phycitis*; and (**I**) *H. paraphycitis*.

**Figure 17:**
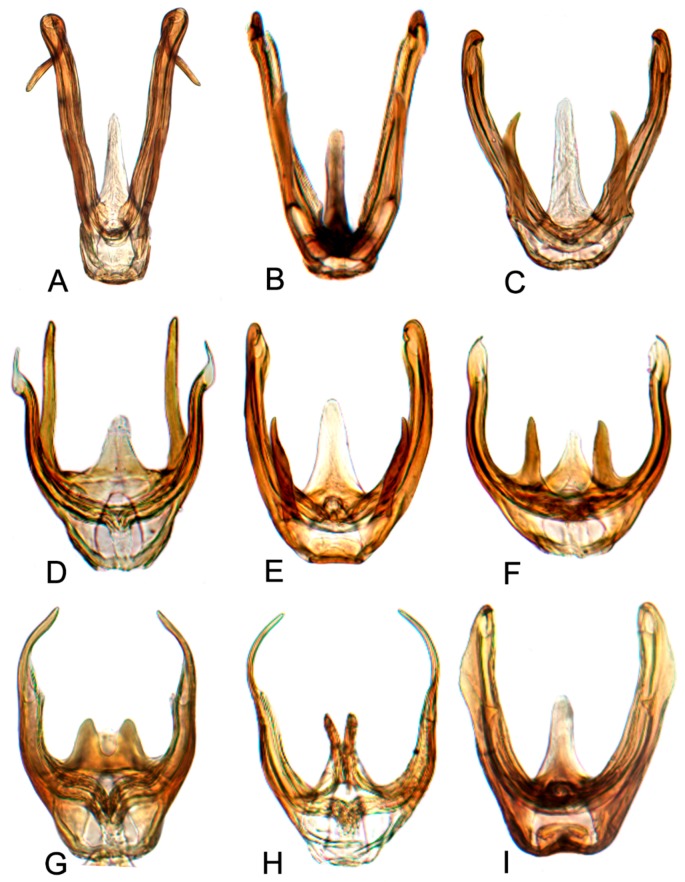
Aedeagus, posterior view. (**A**) *H. prolongatus*; (**B**) *H. spiniferous*; (**C**) *H. kuohi*; (**D**) *H. tortuosus*; (**E**) *H. aberrans*; (**F**) *H. knightiellus*; (**G**) *H. tenuis*; (**H**) *H. subtilis*; and (**I**) *H. diffractus*.

**Figure 18:**
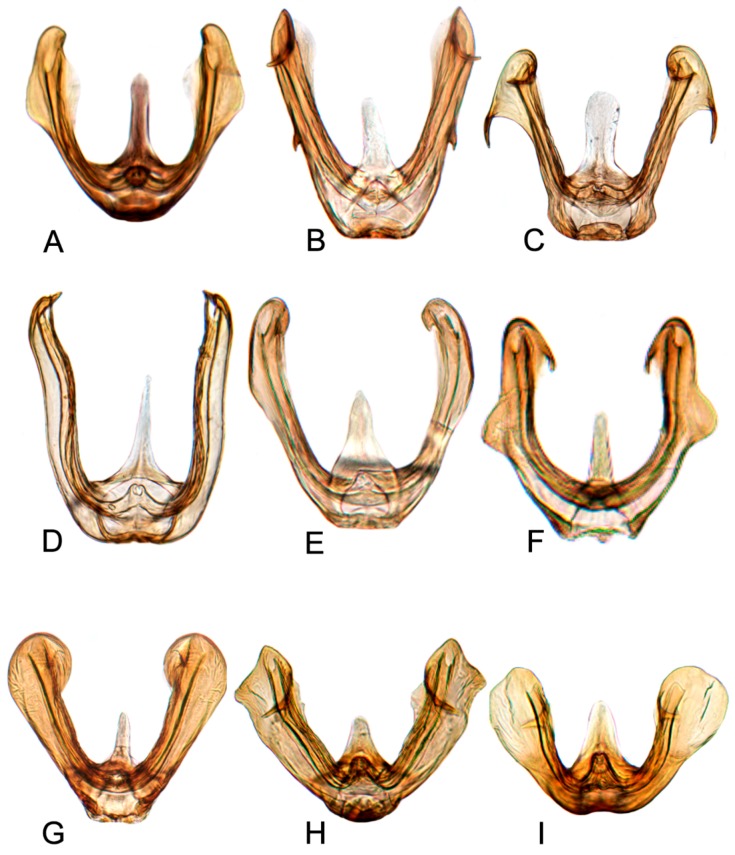
Aedeagus, posterior view. (**A**) *H. expansivus*; (**B**) *H. ventralis*; (**C**) *H. hamuleus*; (**D**) *H. yuanmouensis*; (**E**) *H. hamatus*; (**F**) *H. fuscomaculatus*; (**G**) *H. lii*; (**H**) *H. phycitis*; and (**I**) *H. paraphycitis*.

**Figure 19:**
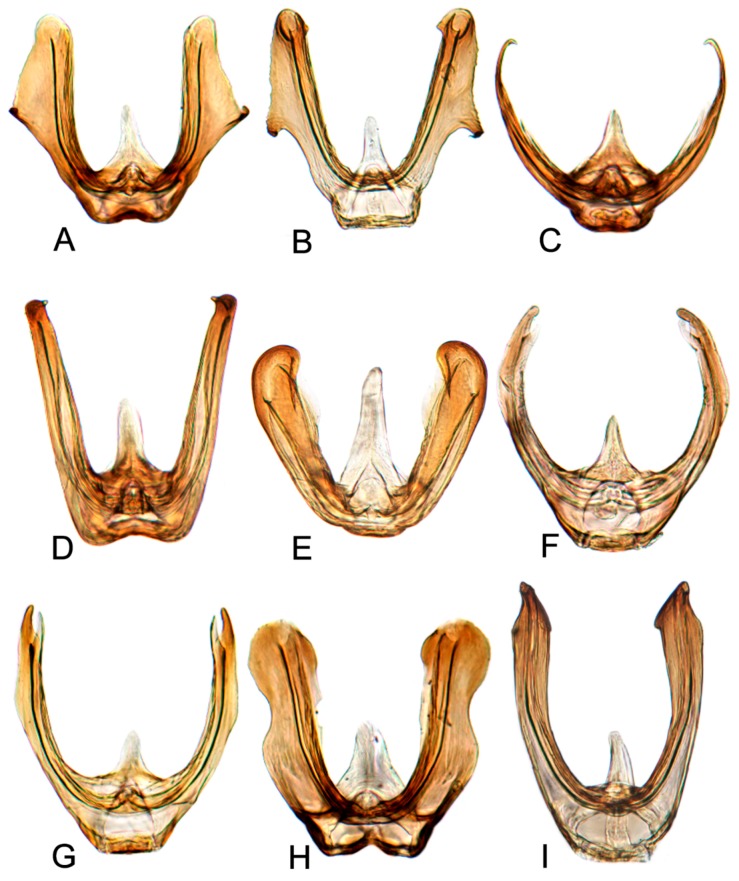
Aedeagus, posterior view. (**A**) *H. viraktamathiellus*; (**B**) *H. triangulus*; (**C**) *H. concavus*; (**D**) *H. dietrichi*; (**E**) *H. bucephalus*; (**F**) *H. fletcheri*; (**G**) *H. dichotomous*; (**H**) *H. sellatus*; and (**I**) *H. rectus*.

**Figure 20:**
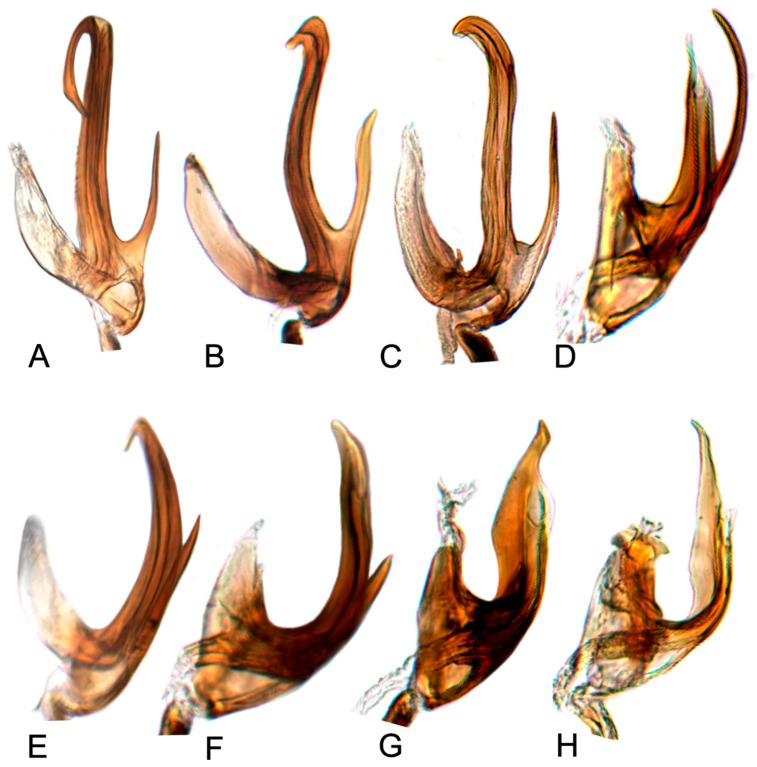
Aedeagus, lateral view. (**A**) *H. prolongatus*; (**B**) *H. spiniferous*; (**C**) *H. kuohi*; (**D**) *H. tortuosus*; (**E**) *H. aberrans*; (**F**) *H. knightiellus*; (**G**) *H. tenuis*; and (**H**) *H. subtilis*.

**Figure 21:**
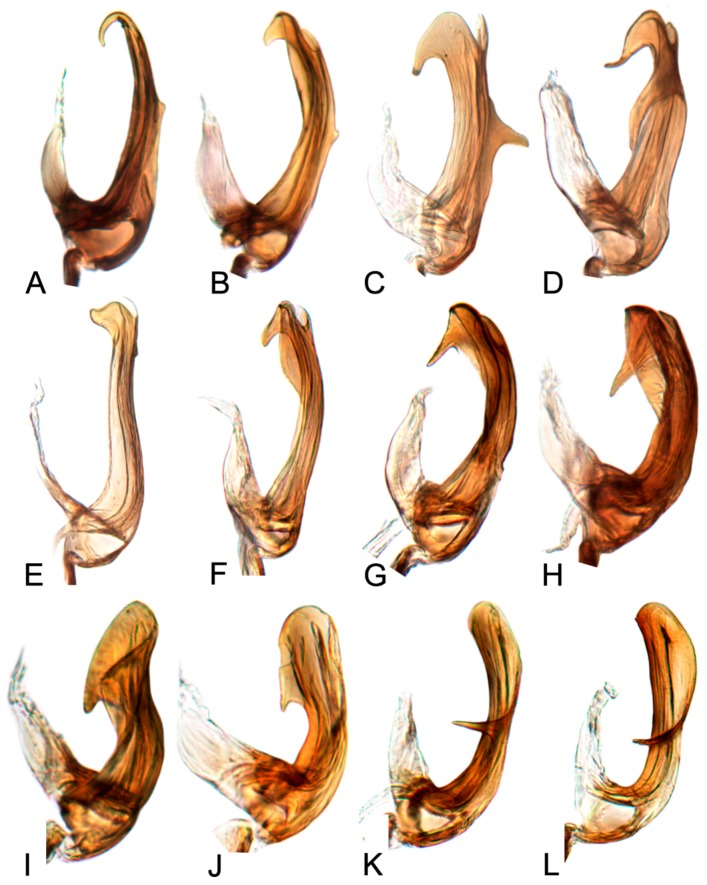
Aedeagus, lateral view. (**A**) *H. diffractus*; (**B**) *H. expansivus*; (**C**) H. ventralis; (**D**) *H. hamuleus*; (**E**) *H. yuanmouensis*; (**F**) *H. hamatus*; (**G**) *H. fuscomaculatus*; (**H**) *H. lii*; (**I**) *H. phycitis*; (**J**) *H. paraphycitis*; (**K**) *H. viraktamathiellus*; and (**L**) *H. triangulus*.

**Figure 22:**
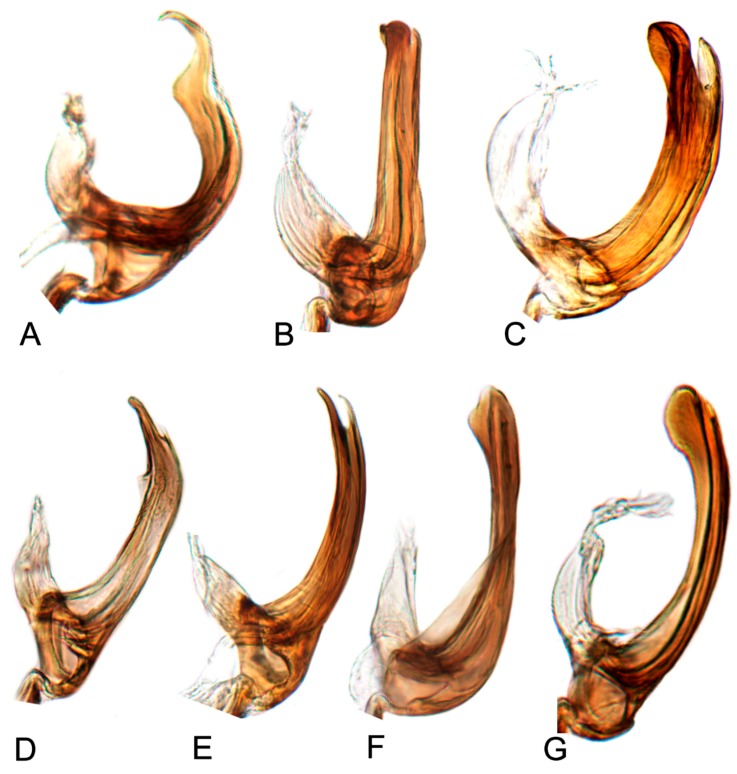
Aedeagus, lateral view. (**A**) H. concavus; (**B**) *H. dietrichi*; (**C**) *H. bucephalus*; (**D**) *H. fletcheri*; (**E**) *H. dichotomous*; (**F**) *H. sellatus*; and (**G**) *H. rectus*.

Figure 4A–C, Figure 9D, Figure 12D, Figure 15D, Figure 18D and Figure 21E.

**Description.** Length: ♂ 4.1 mm. Vertex, face, and pronotum greenish yellow. Scutellum pale brownish yellow with basal triangles delineated by brown margin. Forewings silvery white with yellow brown mottling between veins, becoming darker and dense apically; median diamond patch brown.

**Male genitalia.** Pygofer longer than height in lateral view, posterior margin slightly acute rounded. Valve regularly triangular, with middle length longer than 1/2 basic width. Subgenital plates broadly rounded at base and then margins gradually convex to posterior finger-like process. Style with preapical lobe developed, apophysis long and slender, apex acutely. Connective with arms divergent broadly, equal to stem. Aedeagus with shafts diverged and slightly sinuate in posterior view, with apex recurved inwards; in lateral view, aedeagal shafts slightly sinuate and concaved ventrally at midlength, gradually narrowed to round apex, with apical process reflexed dorsally; gonopores subapical.

**Female.** Unknown.

**Material examined.** Holotype: ♂, Yunnan, Yuanmou, 27.vi. 2005, Li Qiao (NWAFU).

**Distribution.** China (Yunnan).

**Etymology.** The species name refers to the type locality.

**Remarks.** This new species can be identified by the slightly sinuate aedeagal shafts as well as the dorsally inflexed apical process.

#### 3.2.14. *Hishimonus hamatus* Kuoh, 1976

Figure 4D–F, Figure 9E, Figure 12E, Figure 15E, Figure 18E, Figure 21F, Figure 23J and Figure 25B1–B3.

*Hishimonus hamatus* Kuoh, 1976: 432, Figure 3a–d [11]; Li, 1987: 303: Figure 151 [41]; Li & Wang, 1991: 201 [42]; Cai & Shen, 1999: 244 [47]; Cai & Huang, 1999: 329, Figures 16–123(A–F) [48]; Cai, He & Gu, 2001: 207 [46]; Li & Wang, 2004: 486 [13]; Li, Dai & Xing, 2011: 93, Figures 5–82(1–5) [10]; and Seljak, 2013: 124, Figures 1–10 [39].

*Hishimonus araii* Okada, 1978: 308, Figures 1–7 [26].

**Description.** Length: ♂ 3.5–4.2 mm, ♀ 3.5–4.4 mm. Head pale greenish yellow with a pair of anterior indistinctly fuscous spots. Pronotum greenish yellow on anterior half while the posterior half is somewhat dark. Scutellum yellowish brown with basal triangles delineated by brown margin, with apex and one spot on each lateral margin ivory. Forewings silvery white with dark brown mottling between veins, becoming darker and denser apically; pale brownish median diamond patch distinct, well-defined by dark brown at anterior margin.

**Male genitalia.** Pygofer in lateral view longer than height, posterior margin rounded. Subgenital plate broadly rounded at base and then margins gradually convex to posterior finger-like process. Style with preapical lobe not developed, apophysis slender and elongate, as long as 0.4 length of style, inner margin slightly concaved. Connective with stem broad, shorter than divergent arms. Aedeagus in posterior view with dorsal apodeme narrow, shaft constricted at base, slightly expanded near subapex, apex rounded with slight medial notch; in lateral view with dorsal apodeme short, as long as one half of shaft, shafts slightly curved dorsally, gradually narrowed to round apex, lateral margin at subapex slightly protruded as lamellar, apical process reflexed anteriorly; gonopores subapical on posterior surface.

**Female.** Sternite VII nearly twice as wide as long, posterior margin broad V-shaped emargination with a small median projection. Second valvulae with dorsal margin broadly arcuate, teeth prominent.

**Material examined.** 1♂, Fujian, Wuyishan, vii.2010, Han Juan; 14♂♂4♀♀, Guizhou, Kaili, 15.vii.2018, Liang Zonglei; 1♂1♀, Guizhou, Kaili, 29.vii.2016, Wen Chao; 2♂, Guizhou, Bijie, 8.vii.2018, Liang Zonglei; 2♂♂, Guizhou, Tongren, 26.vii.2018, Liang Zonglei; 1♂, Guizhou, Maolan, 4.viii.2016, Wen Chao; 1♂, Guizhou, Fodingshan, 22.vii.2018, Liang Zonglei; 1♂1♀, Guizhou, Leigongshan, 17.vii.2018, Liang Zonglei; 5♂♂, Hubei, Dabieshan, vi.2014, Chen Fangyin; 2♂♂2♀♀, Hunan, Huangsang, 5.vii.2016, Wen Chao; 1♂, Jiangxi, Lushan, 9. ix.2005, Lin Yujian; 1♂, Jiangxi, Zixi, 12.vii.2018, Hu Yulin; 1♂, Shaanxi, Yangling, 23.vi.1985; 5♂♂3♀♀, Sichuan, Jiajiang, vii.2015; 1♂2♀♀, Sichuan, Yaan, 20.vii.2009, Zhang Xinmin; 5♂♂1♀, Zhejiang, Kaihua, 25.viii.2017, Hu Yulin; 3♂♂1♀, Zhejiang, Quzhou, 7.vii.2017, Wang Yan; 2♂♂1♀, Zhejiang, Jiangshan, 3.vii.2017, Wang Yan; 3♂♂2♀♀, Zhejiang, Tianmushan, 26.vi.2018, Hu Yulin; 2♂♂, Zhejiang, Gutian, 17.viii.2003, Dai Wu; and 1♂, Zhejiang, Fengyangshan, 2.viii.2007, Qiao Luman.

**Distribution.** China (Fujian, Guizhou, Henan, Hunan, Jiangxi, Ningxia, Shaanxi, Sichuan, Xinjiang, Zhejiang), Japan, Korea, Slovenia.

**Remarks.***H. hamatus* was first described by Kuoh [11] based materials collected in southern China, and then by Okada [26] in Japan under the name of *H. araii*. The later was recently recognized as a junior synonym [39]. Recently, Seljak [39] recorded this species in Nova Gorica and its surroundings in western Slovenia, which was considered the result of a secondary introduction. This species is very similar to *H. lindbergi* Knight and *H. callisto* Knight, but can be distinguished by the much slenderer shafts of aedeagus with convex outer margins. The female seventh sternite has the posterior margin concave medially with an emarginate black process.

#### 3.2.15. *Hishimonus fuscomaculatus* Li & Wang, 2004

Figure 4G–I, Figure 9F, Figure 12F, Figure 15F, Figure 18F, Figure 21G, Figure 23K and Figure 25C1–C3.

*Hishimonus fuscomaculatus* Li & Wang, 2004: 488, Figures 7–12 [13]; Xing & Li, 2010 [43]: 134; and Li, Dai & Xing, 2011: 92, Figures 5–81(1–6) [10].

**Description.** Length: ♂ 2.9–4.4 mm, ♀ 3.0–4.1 mm. Vertex ochraceous with irregular brown markings. Face ochraceous, with many brown spots. Pronotum ochraceous on anterior half while the posterior half somewhat dark. Scutellum ochraceous with basal triangles and apical half orange brown, with apex and one spot on each lateral margin ivory. Forewings silvery white with dark brown mottling between veins, becoming darker and more dense apically; brown median diamond patch distinct, well-defined by dark brown at anterior margin and posterior corner, with median section anterior to posterior translucent whitish.

**Male genitalia.** Pygofer longer than height in lateral view, posterior margin slightly rounded. Valve is broad triangle with middle length longer than 1/2 basic width. Subgenital plates broadly rounded, and margins lightly convex to elongate posterior finger-like process. Style with poorly developed preapical lobe, apophysis slender, tapering to apex inner margin slightly concave. Connective with stem shorter than arms. Aedeagus with shafts diverged and slightly curved inwards in posterior view forming a hook processes apex, with an auriform processes at outer margin of the midlength; in lateral view, aedeagal shafts curved anteriorly with apical thin process reflexed anteriorly; gonopores subapical on posterior surface.

**Female.** Sternite VII about 2.5 times as wide as long medially, posterior margin deeply V-shaped emargination with two median denticles. Second valvulae with dorsal margin broadly arcuate, teeth prominent.

**Material examined.** 1♂1♀, Shaanxi, Tongtianhe, 25.viii.2015; 3♂♂, Shaanxi, Tongtianhe, 24.viii.2015.

**Distribution.** China (Guizhou, Shaanxi).

**Notes.** This species was described based on two male and three female specimens deposited in the collection of Guizhou University. The illustrations given by Li and Wang [13] were misleading. Dr. J.C. Xing checked the type specimen and (personal communication) confirmed that the male genitalia illustrated here agreed with the male genitalia of the holotype. This species is similar to *H. hamatus* Kuoh, sharing the spoon-like expanded aedeagal shafts, but it can be identified by the auriform processes on the outer margin at midlength.

#### 3.2.16. *Hishimonus lii* Du & Dai, sp. nov.

Figure 4J–L, Figure 9G, Figure 12G, Figure 15G, Figure 18G and Figure 21H.

**Description.** Length: ♂ 4.4 mm. Vertex, face, and pronotum greenish yellow, with indistinct fuscous spots. Scutellum greenish yellow with apical half dark brown. Forewings silvery gray with densely dark brown mottling between veins, becoming darker and more dense basal and apically; dark brown median diamond patch without distinct margins.

**Male genitalia.** Pygofer in lateral view slightly longer than height, posterior margin bluntly rounded. Subgenital plates broadly rounded at base, then margins lightly curved and narrowing to elongate posterior finger-like process. Style with preapical lobe moderate developed, apophysis long, tapering. Connective with stem broad, slightly longer than arms. Aedeagus with shafts moderately divergent in posterior view, with widened elongate basal process from posterior edge of each shaft extending to about half length of shafts. In lateral view, shafts concaved dorsally and gradually narrowing to apex, with apical process reflexed anteriorly; gonopore subapical on posterior surface.

**Female.** Unknown.

**Material examined.** Holotype: ♂, Gansu, Wenxian, 29.vii.2004 (NWAFU).

**Distribution.** China (Gansu).

**Etymology.** The species is named after Prof. Zizhong Li of Guizhou University in recognition of his contributions to leafhopper taxonomy.

**Remarks.** This species is similar to *H. bucephalus* Emeljanov, but it can be identified by the relatively short style and the spoon-like apex of the aedeagal shafts with two pairs of hooked processes.

#### 3.2.17. *Hishimonus phycitis* (Distant, 1908)

Figure 5A–C, Figure 9H, Figure 12I, Figure 15H, Figure 18H, Figure 21I, Figure 23L and Figure 25D1–D3.

*Eutettix phycitis* Distant, 1908: 363, Figure 231 [21].

*Eutettix lugubris* Distant, 1918: 60 [49]. Synonymized by Knight 1970: 128 [9].

*Hishimonus orientalis* Emeljanov, 1969: 1102 [50]. Synonymized by Knight 1970: 128 [9].

*Hishimonus phycitis* Knight 1970: 128, Figures 10, 11, 13 [9]; Kuoh, 1976: 8, Figure 4 [11]; Li, 1987: 303, Figures 152, XXIX-266 [41]; Li & Wang, 1991: 200 [42]; Cai & Huang, 1999: 328, Figures 16–121(A–C) [48]; Zhang, et al, 2004: 205 [51]; Li & Wang, 2004: 486 [13]; Li, Zhang & Wang, 2007: 157 [52]; Xing & Li, 2010: 133 [43]; Li, Dai & Xing, 2011: 94, Figures 5–83(1–5) [10]; Dai, Fletcher & Zhang, 2013: 308, Figures 2A, 2D, 6A–F [7]; Viraktamath & Murthy, 2014: 114, Figures 23–26, 161–176 [8]; Jeger M, et al, 2017: 13 [53]; and Hassan & Zhang, 2018: 1805, Figures 1A–B, 2A–J [40].

**Description.** Length: ♂ 3.4–3.8mm, ♀ 3.5–4.2mm. Head and pronotum greenish yellow, scutellum greenish yellow, with basal triangles and apical half marked with dark brown margins. Forewings silvery white with densely brown mottling between veins; median diamond patch dark brown, with median section translucent whitish.

**Male genitalia.** Subgenital plates broadly rounded at base, evenly rounded to posterior finger-like process. Style with apophysis short, curved slightly posteriorly. Connective with arms slightly shorter than stem. Aedeagus with shafts widely divergent, broad throughout, apically obliquely truncate with apical enlarged posteromedial lobe.

**Female.** Female seventh sternite 2.5 times as wide at base as long medially, posterior margin curved, with a median projection, posterolateral angles acutely rounded. First valvulae about 0.66 as wide as long. Second valvulae with dorsal teeth short.

**Material examined.** 2♂♂2♀♀, Guangdong, Zhaoqing, 20.vii.2017, Du Lan; 2♂♂, Guangdong, Zhaoqing, 17.vi.1983, Zhang Yalin; 1♂, Guangxi, Shangsi, 10.vi.2000, Li Wenshu; 1♂, Guangxi, Dianbai, 12.iv.1983, Zhang Yalin;1♂, Guangxi, Huaping, 29.vii.2017, Du Lan; 1♂, Guangxi, Fangcheng, 1.xii.2001, Wang Zongqing; 1♂1♀, Guizhou, Maolan, 2.viii.2016, Wen Chao; 2♂♂, Hainan, Liangyuan, 1.vi.1983, Zhang Yalin; 1♂, Hainan, Yacheng, 10.v.1983, Zhang Yalin; 3♂♂3♀♀, Hainan, Diaoluoshan, 21.iv.2018, Du Lan; 30♂♂38♀♀, Hainan, Limushan, 11.v.2018, Du Lan; 3♂♂2♀♀, Yunnan, Honghezhou, 13.vii.2017, Liang Zonglei; 1♂2♀♀, Yunnan, Puer, 18.vii.2017, Liang Zonglei; and 1♂, Yunnan, Yuxi, 7.viii.2017, Liang Zonglei.

**Distribution.** China (Fujian, Guangdong, Guizhou, Hainan, Jiangsu, Jiangxi, Macao, Sichuan, Taiwan, Yunnan), Australia, India, Iran, Malaysia, Oman, Pakistan, Philippines, Sri Lanka, Thailand, and United Arab Emirates.

**Remarks.** Coloration of *H. phycitis* including the development of the median spot varies considerably among specimens. It can be identified by the short, broadly lamellate aedeagal shafts with the apex broad and with a lateral spine, and gonopores subapical on the posterior surface. Females have the posterior margin of the seventh sternite concave medially with a black process present.

**Notes.***Hishimonus phycitis* is a tropical and subtropical species that was first described from India, placed in the genus *Eutettix* by Distant [21], and assigned to the genus *Hishimonus* by Nielson [25]. This species occurs in tropical and subtropical Asia from Iran to Malaysia [30]. It was erroneously reported as occurring in Australia by Metcalf [54], Datta [55], and Knight [35]. This species is a vector of little leaf phytoplasma of Brinjal (eggplant).

#### 3.2.18. *Hishimonus paraphycitis* Du & Dai, sp. nov.

Figure 5D–F, Figure 9I, Figure 12I, Figure 15I, Figure 18I, Figure 21J, Figure 23M and Figure 25E1–E3.

**Description.** Length: ♂ 3.4 mm, ♀ 3.6 mm. Vertex, face, and pronotum stramineous with a pair of indistinctly brown spots on vertex. Scutellum yellowish stramineous with basal triangles and apical half brownish yellow, apex and one spot on lateral margins ivory. Forewings silvery white with light brown mottling between veins, becoming darker and more dense apically; brown median diamond patch without distinct margin, with median section translucent whitish.

**Male genitalia.** Pygofer with height equal to length in lateral view, posterior margin rounded. Valve likes broadly triangular, approximately 1/2 basic width as long medically. Subgenital plates broadly rounded at base, and then margins lightly convex to elongate posterior finger-like process. Style with poorly developed preapical lobe, apophysis short and robust. Connective with stem longer than arms. Aedeagus with shafts moderately diverged, almost obtusely extended from midlength towards round apex, with a process at the inner margin; in lateral view, shafts slightly curved anteriorly with small spine process directed apically; gonopores subapical.

**Female.** Female seventh sternite 2.5 times as wide at base as long medially, posterior margin slightly curved, with two small median projections separated by a tiny notch. Second valvulae with dorsal teeth short.

**Material examined.** Holotype: ♂, Hainan, Bawangling, 25.v.1983, Zhang Yalin; Paratypes: 9♀, data same as holotype (NWAFU).

**Distribution.** China (Hainan).

**Etymology.** The species name alludes to the overall similarity of this species to *H. phycitis*.

**Remarks.** This species is similar to *H. phycitis* (Distant), but it can be identified by the circularly expanded aedeagal shaft apices.

#### 3.2.19. *Hishimonus viraktamathiellus* Du & Dai, sp. nov.

Figure 5G–I, Figure 10A, Figure 13A, Figure 16A, Figure 19A, Figure 21K, Figure 23N and Figure 25F1–F3.

**Description.** Length: ♂ 3.3–3.6 mm, ♀ 3.5–3.7 mm. Vertex greenish yellow, with a pair of indistinct brown spots apically. Face and pronotum greenish yellow. Pronotum greenish yellow on anterior half while the posterior half somewhat dark. Scutellum brownish yellow with dark brown spots on each side of lateral margin and anterior side of transverse impressed line, apex and a spot on each lateral margin white. Forewings silvery white with dark brown veins as well as the dense dark brown mottling between veins, becoming darker and more dense apically; median diamond patch indistinct, with median section translucent whitish.

**Male genitalia.** Pygofer longer than height in lateral view, posterior margin rounded. Valve triangular, middle length slight longer than 1/2 basic width. Subgenital plates with lateral margins convex at base, then lightly curved before narrowing to elongate posterior finger-like process. Style with preapical lobe undeveloped, apophysis short, slightly curved laterally. Connective with stem longer than arms. Aedeagus with shafts moderately diverged and being widest at midlength where the outer margin expanded triangularly in posterior view; in lateral view, shafts slightly curved dorsally, with ventral margin slightly widened subapically, a spine-like processes directed anteriorly at basal 2/5; gonopores apical.

**Female.** Sternite VII nearly twice as wide as long, posterior margin broad V-shaped emargination with a small medial lobe. Second valvulae with dorsal teeth large and prominent, conical.

**Material examined.** Holotype: ♂, Hainan, Yinggeling, 5.v.2018, Du Lan; Paratypes: 8♂♂1♀, data same as holotype; and 1♂1♀, Hainan, Diaoluoshan, 21.iv.2018, Du Lan (NWAFU).

**Distribution.** China (Hainan).

**Etymology.** The species is named after Prof. Chandra Viraktamath in recognition of his contributions to leafhopper taxonomy.

**Remarks.** The new species is similar to *H. triangulus* sp. nov., but the later has a pair of small hook-like processes at the base of aedeagus and the midlength shaft process longer.

#### 3.2.20. *Hishimonus triangulus* Du & Dai, sp. nov.

Figure 5J–L, Figure 10B, Figure 13B, Figure 16B, Figure 19B, Figure 21L, Figure 23O and Figure 25G1–G3.

**Description.** Length: ♂ 3.3–3.6 mm, ♀ 3.5–3.9 mm. Vertex pale greenish yellow, with anterior marginal and submarginal faint spots. Face greenish yellow, with faint submarginal spots. Pronotum greenish yellow on anterior half while the posterior half somewhat dark. Scutellum greenish yellow, with basal triangles and apical half delineated by brown margin, apex and one spot on each side of anterior margin white. Forewings silvery white with sparse slightly brown spots between brown veins, becoming darker and more dense apically; brown median diamond patch distinct, surrounded by dark brown at anterior margin, with median section anterior to posterior translucent whitish.

**Male genitalia.** Pygofer slightly higher than length in lateral view, posterior margin blunt rounded. Valve triangular, obviously longer than 1/2 basal width. Subgenital plates broad at base, with lateral margins roundly curved before narrowing to elongate posterior finger-like process. Style with preapical lobe developed, apophysis straight and slender, tapering to apex. Connective with stem slightly longer than arms. Aedeagus in posterior view with shafts moderately diverged and apical half expanded laterally, and then tapering to spine-like process directed anteriorly at the middle, with a small apical tooth at inner margin; in lateral view shaft with dorsal margin straight, ventral margin widened subapically, with a spine-like process directed anteriorly at basal 1/3; gonopores apical.

**Female.** Sternite VII nearly 2.5 times as wide as long, posterior margin broad V-shaped emargination with two medial denticles. Second valvulae with dorsal margin broadly arcuate, teeth large and prominent, irregularly spaced.

**Material examined.** Holotype: ♂, Zhejiang, Quzhou, 28.viii.2017, Hu Yulin; Paratypes: 1♂7♀♀, date same as holotype; 1♂, Hunan, Huangsang, 5.vii.2016, Wen Chao; 9♂♂1♀, Hunan, Hengshan, 3.viii.1985, Zhang Yalin & Chai Yonghui; 39♂♂7♀♀, Hunan, Chenzhou, 3.viii.1985, Zhang Yalin & Chai Yonghui; and 2♂♂, Zhejiang, Tianmushan, 26.vii.2011, Wang Yang (NWAFU).

**Distribution.** China (Zhejiang, Hunan).

**Etymology.** The species name refers to aedeagal shafts expanded triangularly.

**Remarks.** This species is similar to *H. gillespiei* Dai, Fletcher & Zhang, but the latter has obvious anterior hooks towards the shaft apex.

#### 3.2.21. *Hishimonus concavus* Knight, 1970

Figure 6A–C, Figure 10C, Figure 13C, Figure 16C, Figure 19C, Figure 22A, Figure 23P and Figure 26A1–A3.

*Hishimonus concavus* Knight, 1970: 133, Figures 36–38 [9]; Kuoh, 1976: 8, Figure 7 [11]; Li & Wang, 2004: 487 [13]; Li, Dai & Xing, 2011: 90, Figures 5–78(1–5) [10]; Dai, Fletcher & Zhang, 2013: 303, Figures 1A, 1D, 3A–F [7]; and Viraktamath & Murthy, 2014: 108, Figures 7–8, 72–79 [8].

**Description.** Length: ♂ 2.5–3.5mm, ♀ 3.4–3.8mm. Vertex, face and pronotum pale greenish yellow. Scutellum brown yellow marked with brown mottling. Forewings silvery gray with densely dark brown mottling; brown median diamond patch indistinct, with median section anterior to posterior translucent whitish.

**Male genitalia.** Pygofer in lateral view longer than height, posterior margin conically rounded. Valve about twice as wide at base as long. Subgenital plate broadly rounded laterally at base then tapered to apical point bearing terminal finger-like process. Style approximately twice as long as wide at base; preapical lobe well developed with a few hair-like setae, apophysis short and straight, slightly directed posteriorly. Connective with stem slender, as long as arms. Aedeagus with shafts widely separated at base, curving dorsally with apex of each shaft recurved; in lateral view, shafts curved dorsally, abruptly narrowed subapically and tapering to apex reflexed anteriorly, with a flange on anterior margin in basal half; gonopores nearly midlength on posterior surface.

**Female.** Sternite VII nearly twice as wide at base as long medially, posterior margin truncates, with two median denticles. Second valvulae with dorsal teeth short and widely spaced.

**Material examined.** 2♂♂6♀♀, Hainan, Bawangling, 25.v.1983, ZhangYalin.

**Distribution.** China (Fujian, Guangdong, Hainan, Taiwan), India, Philippines, Thailand.

**Remarks.** Knight’s [9] original material comprised only two males and four females from two localities in the Philippines. This species is more widespread in Southeast Asia. Knight [9] considered its affinities to be with *H. arcuatus* Knight from Sri Lanka, which is similar in the structures of the aedeagus, connective, and style but differs in having a short tooth-like process rather than a lamellate expansion on each shaft.

#### 3.2.22. *Hishimonus dietrichi* Du & Dai, sp. nov.

Figure 6D–F, Figure 10D, Figure 13D, Figure 16D, Figure 19D, Figure 22B, Figure 23Q and Figure 26B1–B3.

**Description.** Length: ♂ 2.5–3.5 mm, ♀ 3.4–3.8 mm. Vertex, face and pronotum greenish yellow. Scutellum brownish yellow with dark brown mottling, apex and one spot on each side of anterior margin white. Forewings silvery white with brown mottling between veins, becoming darker and more dense apically; median diamond patch distinct, surrounded by brown at anterior margin and posterior corner, with median section translucent whitish.

**Male genitalia.** Pygofer longer than height in lateral view, posterior margin blunt rounded. Valve is regular triangle with middle length longer than 1/2 basal width. Subgenital plates broadly rounded at base, and then margins lightly curving to elongate posterior finger-like process. Style with preapical lobe poorly developed, apophysis elongate and slender, tapering to apex. Connective with stem broad, approximately equal to arms. Aedeagus with shafts moderately diverged, straightened towards apex in posterior view, with an apical tooth on the inner margin; in later view, shafts straight, slightly narrowed to round apex with a small tooth; gonopores apical.

**Female.** Female seventh sternite 2.5 times as wide at base as long medially, posterior margin slightly curved with two median denticles separated by a tiny notch. Second valvulae with dorsal teeth short and somewhat irregularly spaced.

**Material examined.** Holotype: ♂, Yunnan, Yuxi, 7.viii.2017, Liang Zonglei; Paratype: 3♂♂1♀, data same as holotype; and 1♂1♀, Yunnan, Honghezhou, 10.vii.2017, Liang Zonglei (NWAFU).

**Distribution.** China (Yunnan).

**Etymology.** The species is named after Prof. Chris Dietrich of the Illinois Natural History Survey, University of Illinois at Urbana-Champaign in recognition of his contributions to leafhopper taxonomy.

**Remarks.** This species can be identified by the apical processes on the straight aedeagal shaft.

#### 3.2.23. *Hishimonus bucephalus* Emeljanov, 1969

Figure 6G–I, Figure 10E, Figure 13E, Figure 16E, Figure 19E, Figure 22C, Figure 23R and Figure 26C1–C3.

*Hishimonus bucephalus* Emeljanov, 1969: 1101 [50]; Knight, 1970: 130, Figures 18–19 [9]; Cai & Shen, 1998: 248 [56]; and Cai & Shen, 1999: 244 [47].

*Hishimonus reflexus* Kuoh, 1976: 431, Figure 1 [11]; Li & Wang, 1991: 199, Figure 103 [42]; Cai & Huang, 1999: 327, Figure 16-120(A–I) [48]; Li & Wang, 2004: 486 [13]; and Li, Dai & Xing, 2011: 96, Figures 5–86(1–3) [10]. **syn. nov.**

*Hishimonus biuncinatus* Li, 1988: 52, Figure 2 [12]; Li & Wang, 1991: 202, Figure 105 [42]; Li & Wang, 2004: 487 [13]; Li & Wang, 2006: 186 [57]; Li, Zhang & Wang, 2007: 158 [52]; Xing & Li, 2010: 133 [43]; and Li, Dai & Xing, 2011: 90, Figures 5–77(1–6) [10]. syn. nov.

**Description.** Length: ♂ 3.3–4.6 mm, ♀ 4.0–5.2 mm. Vertex, face, pronotum, and scutellum greenish yellow. Scutellum with basal triangles and apical half delineated by brown margin, apex and one spot on each lateral margin ivory. Forewings silvery white with dense brown mottling between veins, becoming darker and more dense apically; brown median diamond patch indistinct.

**Male genitalia.** Pygofer in lateral view slightly longer than height, posterior margin bluntly rounded. Subgenital plates broadly rounded at base, then lateral margins lightly curved to elongate posterior finger-like process. Style with preapical lobe poorly developed, apophysis elongate. Connective with stem slightly longer than arms. Aedeagus with shafts moderately divergent in posterior view, with slight widening to round apex; in lateral view, shafts curved dorsally; gonopore subapical on posterior surface.

**Female.** Sternite VII twice as wide as long, posterior margin curved, with a median denticle. Second valvulae with dorsal margin almost straight, teeth reduced.

**Material examined.** 13♂♂14♀♀, Fujian, Wuyishan, 27.vii.2013, Feng Ling; 1♂, Fujian, Wuyishan, 17.viii.2008, Gao Xia & Li Xiaoting; 1♂, Guangdong, Dinghushan, 17.vii.1985, Zhang Yalin; 1♂, Guizhou, Huaxi, 25.vii.2001, Sun Qiang; 2♂♂, Henan, Jigongshan, 11.vii.1997, Hu Jian; 2♂♂, Henan, Neixiang, 11.vii.1998, Hu Jian; 2♂♂1♀, Hubei, Yidu, 15.vii.2008, Liu Nian; 3♂♂, Hunan, Hengshan, 8.viii.1985, Zhang Yalin & Chai Yonghui; 1♂, Jiangxi, Zixi, 12.vii.2018, Hu Yulin; 3♂♂, Jiangxi, Yushan, 25.vii.2013, Feng Ling; 1♂, Jiangxi, Anfu, 10.viii.2002, Sun Qinxia; 1♂, Jiangxi, Jinggangshan, 3.viii.2004, Wei Cong & Yang Meixia; 1♂1♀, Shaanxi, Huoditang, 20.vii.2018, Xu Yifei; 1♂, Shaanxi, Huoditang, 15.vii.2004, Wang Shuna; 2♂♂, Shaanxi, Taibaishan, 15.vii.2002, Dai Wu; 4♂♂, Shanxi, Yuanqu, 18.vii.2006, Duan Yani; 1♂, Zhejiang, Jiangshan, 3.vii.2017, Wang Yan; and 1♂, Zhejiang, Linan, 12.viii.2007, Zhang Xinmin.

**Distribution.** China (Fujian, Guangdong, Guizhou, Henan, Hubei, Hunan, Jiangxi, Shaanxi, Shanxi, and Zhejiang) and Russia.

**Remarks.** Based on the descriptions provided by Emeljanov [50] and Kuoh [11], two new synonyms are proposed here, *Hishimonus reflexus* Kuoh, 1976 syn. nov. and *Hishimonus biuncinatus* Li, 1988 syn. nov. This species has each aedeagal shaft broadly rounded at the apex with a hood-like expansion anteriorly in posterior view and the gonopore subapical on the posterior surface. The female seventh sternite has the posterior margin concave medially with a small medial black process.

#### 3.2.24. *Hishimonus fletcheri* Du & Dai, sp. nov.

Figure 6J–L, Figure 10F, Figure 13F, Figure 16F, Figure 19F, Figure 22D, Figure 23S and Figure 26D1–D3.

**Description.** Length: ♂ 3.5–4.1 mm, ♀ 3.5–3.6 mm. Vertex light yellow with indistinct brown spots apically and posteriorly. Face light yellow, with faint arched lines. Pronotum greenish yellow with several indistinct fuscous spots on anterior margin. Scutellum brownish yellow with basal triangles and apical half delineated by brown margin, apex and one spot at each side of anterior margin white. Forewings silvery white with dark brown mottling between veins, becoming darker and more dense apically; brown median diamond patch distinct, surrounded by dark brown at anterior margin, with median section anterior to posterior translucent whitish.

**Male genitalia.** Pygofer longer than height in lateral view, posterior margin slightly angled. Valve is broad triangle with middle length longer than 1/2 basic width. Subgenital plates broadly rounded at base, and then margins slightly narrowing to elongate posterior finger-like process. Style with poorly developed preapical lobe; posterior process is slender, tapering to midlength of aedeagus. Stem of connective slightly longer than arms. Aedeagus with shafts diverged and flexed towards apex in posterior view, with a subapical fork. In lateral view, shafts straight at basal 2/3, then abruptly curved dorsally and narrowing to apex, with a small lamellar expanded at subapex on dorsal margin; gonopores nearly subapex on posterior surface where shaft abruptly tapers.

**Female.** Female seventh nearly 2.5 times as wide at base as long medially, posterior margin truncates, with a tiny projection. Second valvulae with dorsal teeth short, rounded, and somewhat irregularly spaced.

**Material examined.** Holotype: ♂, Yunnan, Mengyang, 27.vii.2011, Zhang Huining; Paratypes: 6♂♂8♀♀, data same as holotype; and 1♂, Yunnan, Gaoligongshan (NWAFU).

**Distribution.** China (Yunnan).

**Etymology.** The species is named in honor of Prof. Murray J. Fletcher, who has made immeasurable contributions to knowledge of leafhopper taxonomy.

**Remarks.** This species is similar to *H. concavus* Knight, but can be identified by mesally curved aedeagal shafts and the poorly developed preapical lobe of the style.

#### 3.2.25. *Hishimonus dichotomous* Kuoh, 1976

Figure 7A–C, Figure 10G, Figure 13G, Figure 16G, Figure 19G, Figure 22E, Figure 23T and Figure 26E1–E3.

*Hishimonus dichotomous* Kuoh, 1976: 433, Figure 4a–c [11]; Li & Wang, 2004: 487 [13]; and Li, Dai & Xing, 2011: 91, Figures 5–79(1–5) [10].

**Description.** Length: ♂ 3.3–4.0 mm, ♀ 3.5–4.2 mm. Vertex, face, and pronotum greenish yellow, with indistinct brown spots. Face with faint arched lines. Scutellum brownish yellow, with basal triangles and apical half delineated by brown margin, apex and one spot at each side of anterior margin white. Forewings silvery white with little brown spots between veins, becoming darker and slightly dense apically; brown median diamond patch distinct, surrounded by dark brown at anterior margin, with median section anterior to posterior translucent whitish.

**Male genitalia.** Pygofer in lateral view longer than height, posterior margin rounded. Valve about twice as wide at base as long. Subgenital plate broadly rounded at base, and then margins gradually convex to posterior finger-like process. Style with preapical lobe poorly developed, apophysis blunt, gradually tapering to apex. Connective with stem longer than arms. Aedeagus in posterior view with shafts divergent, straight, with two short process at apex; in lateral view, shaft gradually curved anteriorly and gradually tapering to apex; gonopore subapical on posterior surface.

**Female.** Sternite VII twice as wide as long, posterior margin slightly curved, with a tiny medial projection. Second valvulae with dorsal margin biarcuate, dorsal teeth short, more closely set distally.

**Material examined.** 2♂♂2♀♀, Hainan, Yinggeling, 5.v.2018, Du Lan; 2♂♂6♀♀, Yunnan, Ruili, 18.v.2011; 4♂♂2♀♀, Yunnan, Yuanyang, 10.vii.2017, Liang Zonglei; and 2♂♂1♀, Yunnan, Yexianggu, 26.vii.2017, Liang Zonglei.

**Distribution.** China (Hainan, Yunnan).

**Remarks.***H. dichotomous* was described by Kuoh [11] based on one male and one female specimen from Yunnan. It can be identified by the divided shaft apex with gonopores in the crotch on the posterior surface.

#### 3.2.26. *Hishimonus sellatus* (Uhler, 1896)

Figure 7D–F, Figure 10H, Figure 13H, Figure 16H, Figure 19H, Figure 22F, Figure 23U and Figure 26F1–F3.

*Thamnotettix sellata* Uhler, 1896: 294 [58].

*Eutettix sellatus*, Matsumura, 1902: 381 [18].

*Eutettix sellata*, Kirkaldy, 1906: 331 [59].

*Acocephalus sellatus*, Ishihara, 1953: 38 [17].

*Hishimonus disciguttus* Esaki & Ito, 1954: 38 [23].

*Hishimonus sellatus*, Ishihara, 1959:48 [60]; Metcalf, 1967: 713 [54]; Knight, 1970: 128, Figures 1–9 [9]; Kuoh, 1976: 7, Figure 1 [11]; Okada, 1978: 310, Figures 8–14 [26]; Kuoh, 1986: 163 [61]; Li, 1987: 304, Figure 153 [41]; Zhang, 1990: 88, Figure 82 [62]; Li & Wang, 1991: 199, Figure VII-2 [42]; Liang et al., 1997: 345 [63]; Cai, He & Zhu, 1998: 72 [64]; Cai & Shen, 1998: 248 [56]; Cai & Huang, 1999: 328, Figure 16-122(A–F) [48]; Cai & Shen, 2002: 277 [65]; Cai & He, 2002: 134–157 [66]; Li & Wang, 2004: 486 [13]; Li & Wang, 2006: 186 [57]; Li, Zhang & Wang, 2007: 157 [52]; Xing & Li, 2010: 133 [43]; and Li, Dai & Xing, 2011: 97, Figures 5–87(1–5) [10].

**Description.** Length: ♂ 3.7–4.5 mm, ♀ 4.5–5.0 mm. Head, pronotum, and scutellum yellow or greenish yellow marked with pale brown, especially the posterior of pronotum. Forewings silvery white with thin brown mottling between veins, median diamond patch distinct, surrounded by dark brown at anterior margin and a posterior corner, with median section anterior to posterior translucent whitish.

**Male genitalia.** Pygofer longer than height in lateral view, posterior margin slightly angulate. Valve triangular, approximately 1/2 as long medially as wide at base. Subgenital plates rounded at base, and gradually tapered to posterior forming relatively short finger-like processes. Style with developed preapical lobe, apophysis slender. Stem of connective longer than arms. Aedeagus with shafts widely diverged from the base, apex rounded, lateral margin bisinuate in posterior view; in lateral view, shafts slightly curved dorsally and concaved at dorsal margin; gonopores subapical on posterior surface.

**Female.** Sternite VII nearly twice as wide as long, posterior margin slightly curved with two median denticles separated by a tiny notch. Second valvulae with dorsal teeth small, not prominent.

**Material examined.** 2♂♂4♀♀, Shaanxi, Taibai, 21.viii.2017, Dai Wu; 1♂, Shanxi, Changzhi, vii.2010, Cao Yanghui; 2♂♂, Sichuan, Dongshan, vii.2010, Cao Yanghui; and 1♂, Zhejiang, Zhoushan, 30.vi.2017, Wang Yan.

**Distribution.** China (Anhui, Fujian, Guizhou, Henan, Hongkong, Hubei, Hunan, Jiangsu, Jiangxi, Liaoning, Ningxia, Shandong, Shaanxi, Shanxi, Sichuan, Taiwan, Xinjiang, and Zhejiang), Afghanistan, Armenia, Australia, Ethiopia, Georgia, India, Indonesia, Japan, Korea, Malaysia, Papua New Guinea, Philippines, Russia, Sri Lanka, and Tanzania.

**Notes.***H. sellatus* was originally described by Uhler [58] as *Thammotettix sellata* from Japan based on two female specimens that are deposited in the U.S. National Museum. Unfortunately, *Thamnotettix sellata* Uhler was confused with *Acocephalus discigutta* Walker for a long time because of the absence of information on the male genitalia. Ishihara [60] redescribed *Hishimonus sellatus* as a distinct species. Later, Nielson [25], Knight [9], and Okada [26] examined a cotype and redescribed it, respectively. *H. sellatus* can be identified by the short, broadly lamellate, laterally concave aedeagal shafts.

#### 3.2.27. *Hishimonus rectus* Kuoh, 1976

Figure 7G–I, Figure 10I, Figure 13I, Figure 16I, Figure 19I, Figure 22G, Figure 23V and Figure 26G1–G3.

*Hishimonus rectus* Kuoh, 1976: 432, Figures 2a–d [11]; Li & Wang, 2004: 487 [13]; and Li, Dai & Xing, 2011: 96, Figures 5–85(1–5) [10].

**Description.** Length: ♂ 3.5–3.8 mm, ♀ 3.5–4.0 mm. Head silvery white, with indistinct brown spots apically and posteriorly. Pronotum silvery white on anterior half while the posterior half somewhat dark. Scutellum brownish yellow, with basal triangles and apical half delineated by brown margin, apex and one spot at each side of anterior margin white. Forewings silvery white with little brown spots between veins and dense brown mottling apically, brown median diamond patch distinct, surrounded by dark brown at anterior and posterior margins, with median section anterior to posterior translucent whitish.

**Male genitalia.** Pygofer in lateral view longer than its height, posterior margin conically rounded. Valve shorter than its width at base. Subgenital plates outwardly broadly curved to posteriorly abruptly roundly truncate to narrow finger-like process. Style with preapical lobe moderately developed, apophysis short, tapering to apex. Connective with arms about as long as stem. Aedeagus in posterior view with shafts moderately divergent from base, slightly tapering to obliquely truncate apex; in lateral view shafts curved dorsally, abruptly broaden dorsally near apex; gonopore subapical on posterior side.

**Female.** Seventh sternite 2.5 times as wide at base as long medially, posterior margin broad curved, with two tiny denticles, posterolateral angles acutely rounded. First valvulae, in lateral view, tapering to apex. Second valvulae, in lateral view, dorsal margin arcuate with several large teeth.

**Material examined.** 1♂, Anhui, Jingangling, 27.vii.2007; 3♂♂1♀, Guangdong, Yuexiu Park, 15.vii.1985, Zhang Yalin; 1♂1♀, Guangxi, Yizhou; 2♂♂1♀, Guizhou, Chishui, 24.viii.2012, Wang Yang; 1♂, Sichuan, Mabian; and 1♂, Zhejiang, Gutian, 17.viii.2003, Dai Wu.

**Distribution.** China (Anhui, Guangdong, Guangxi, Guizhou, Hainan, Sichuan, Taiwan, and Zhejiang).

**Remarks.***H. rectus* was described by Kuoh [11] based on one male specimen from Guangxi. It can be identified by the lamellar and twisted apex of the aedeagal shaft as well as by the apical gonopore. The female has the posterior margin of the seventh sternite concave medially with the black process slightly emarginated.

#### 3.2.28. *Hishimonus truncatus* Kuoh, 1976

*Hishimonus truncatus* Kuoh, 1976: 433, Figure 5a–c [11]; Cai & Huang, 1999: 329, Figures 16–124(A–E) [48]; and Li & Wang, 2004: 487 [13].

**Distribution.** China (Fujian, Guangdong).

**Remarks.***H. truncatus* was described by Kuoh [11] based on a male and a female specimen from Guangdong. This species is similar to *H. sellatus* (Uhler), but it differs in having the aedeagal shafts with outer margins not concaved. This species also can be distinguished by the truncated aedeagal shaft apices.

## 4. Conclusions

Twenty-eight species of the leafhopper genus *Hishimonus* Ishihara from China are reviewed based on comparative morphological study, including ten new species, two new-recorded species and three new synonymies. The study also improves informations of the geographical distribution of the genus. In this study, aedeagus, the reliable classified character, trends to form two groups by having the basical process or not, which maybe provides new ideas to further research.

## Figures and Tables

**Figure 2:**
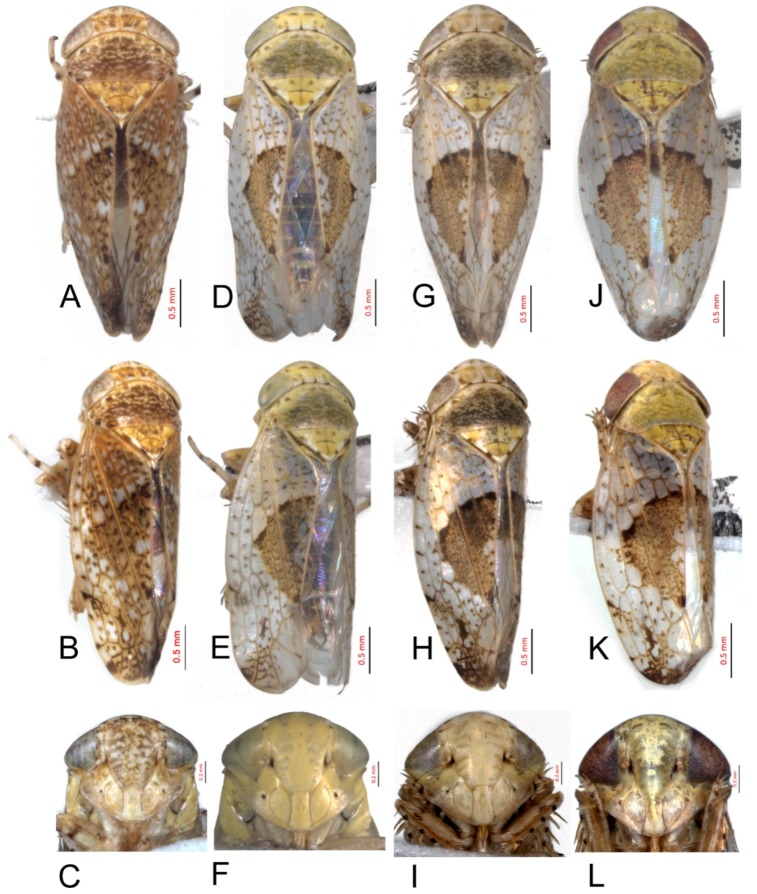
Species of *Hishimonus*. (**A**–**C**) *H. aberrans*; (**D**–**F**) *H. knightiellus*; (**G**–**I**) *H. tenuis*; and (**J**–**L**) *H. subtilis*. (**A**,**D**,**G**,**J**) Dorsal view; (**B**,**E**,**H**,**K**) latero-dorsal view; and (**C**,**F**,**I**,**L**) Face.

**Figure 5:**
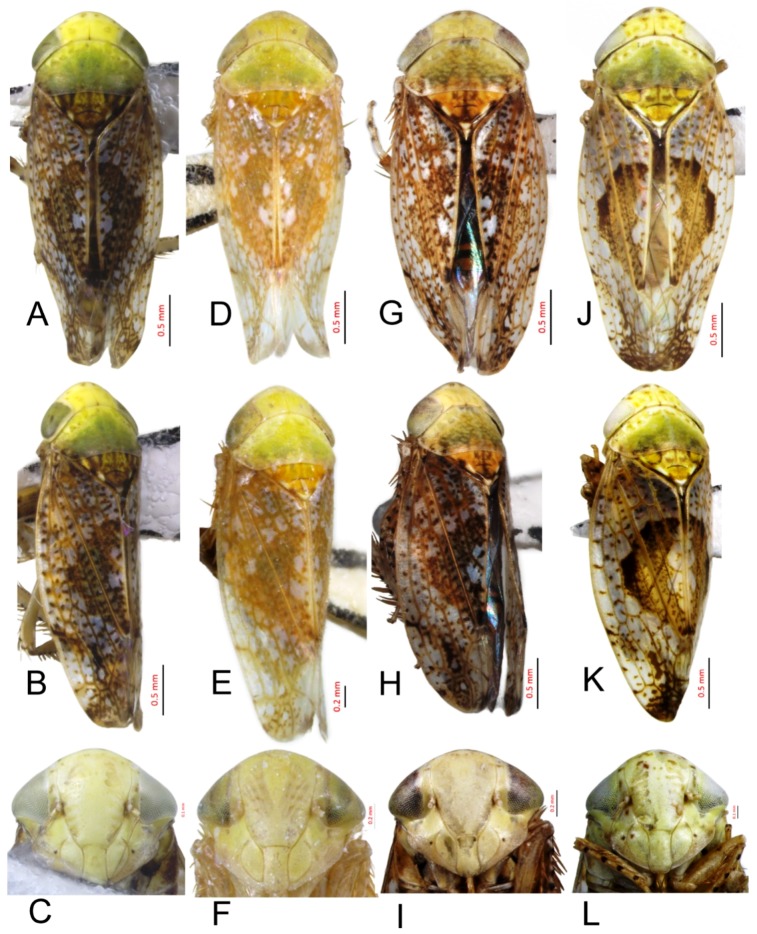
Species of *Hishimonus*. (**A**–**C**) *H. phycitis*; (**D**–**F**) *H. paraphycitis*; (**G**–**I**) *H. viraktamathiellus*; and (**J**–**L**) *H. triangulus*. (**A**,**D**,**G**,**J**) Dorsal view; (**B**,**E**,**H**,**K**) latero-dorsal view; (**C**,**F**,**I**,**L**) Face.

**Figure 6:**
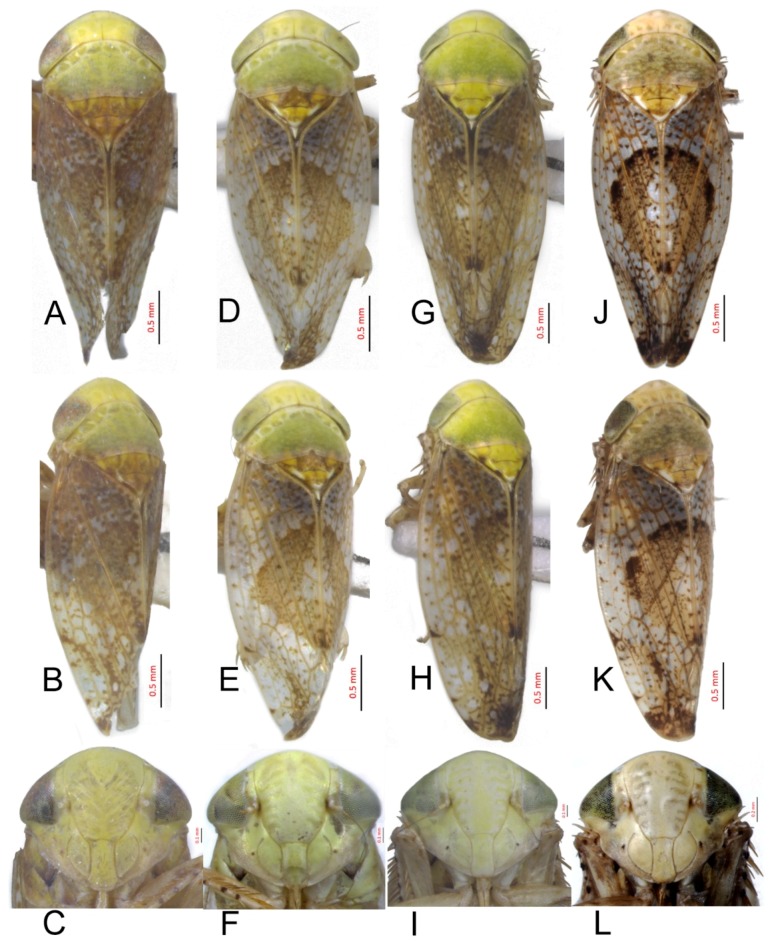
Species of *Hishimonus*. (**A**–**C**) *H. concavus*; (**D**–**F**) *H. dietrichi*; (**G**–**I**) *H. bucephalus*; and (**J**–**L**) *H. fletcheri*. (**A**,**D**,**G**,**J**) Dorsal view; (**B**,**E**,**H**,**K**) latero-dorsal view; (**C**,**F**,**I**,**L**) Face.

**Figure 7:**
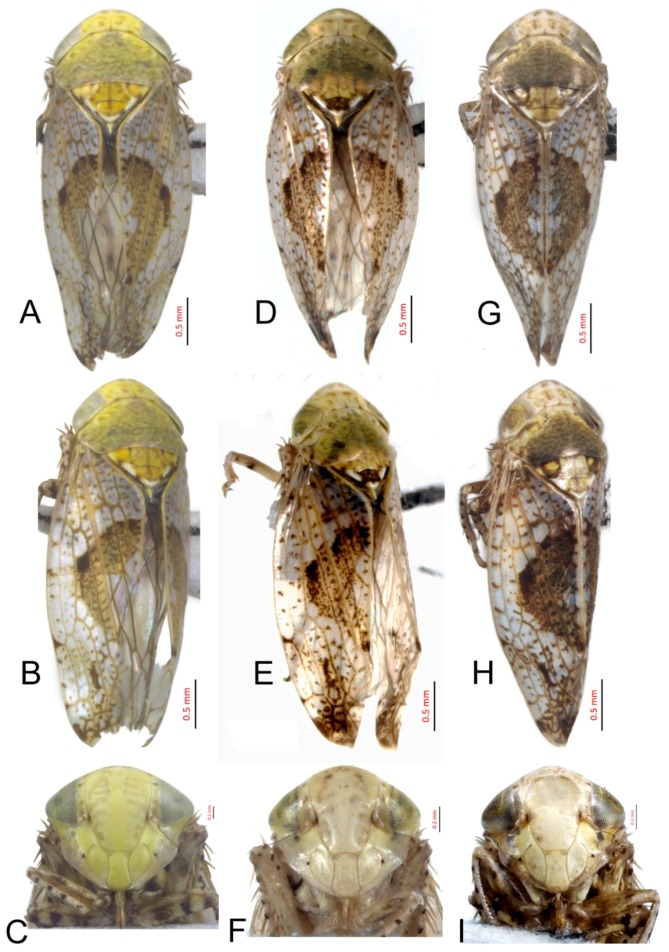
Species of *Hishimonus*. (**A**–**C**) *H. dichotomous*; (**D**–**F**) *H. sellatus*; and (**G**–**I**) *H. rectus*. (**A**,**D**,**G**) Dorsal view; (**B**,**E**,**H**) latero-dorsal view; (**C**,**F**,**I**) Face.

**Figure 10:**
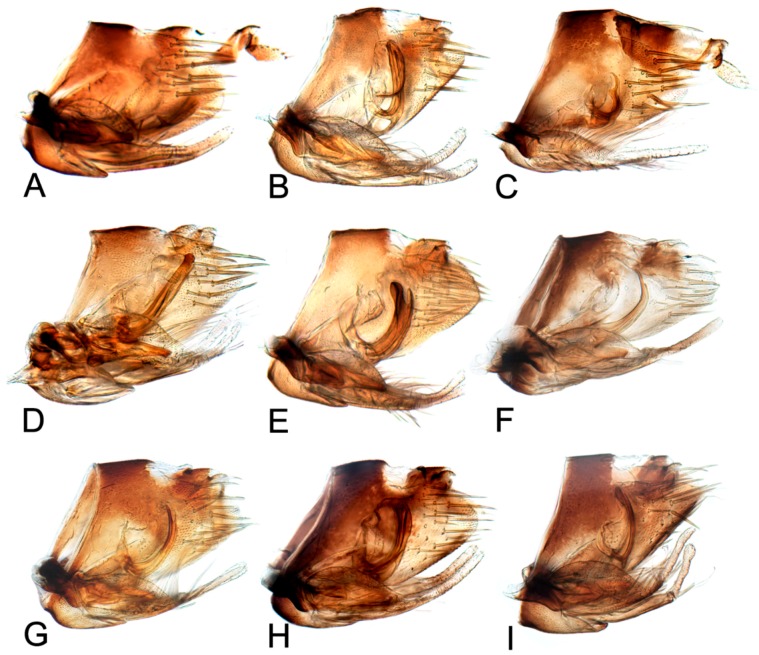
Male pygofer, lateral view: (**A**) *H. viraktamathiellus*; (**B**) *H. triangulus*; (**C**) *H. concavus*; (**D**) *H. dietrichi*; (**E**) *H. bucephalus*; (**F**) *H. fletcheri*; (**G**) *H. dichotomous*; (**H**) *H. sellatus*; and (**I**) *H. rectus*.

**Figure 13:**
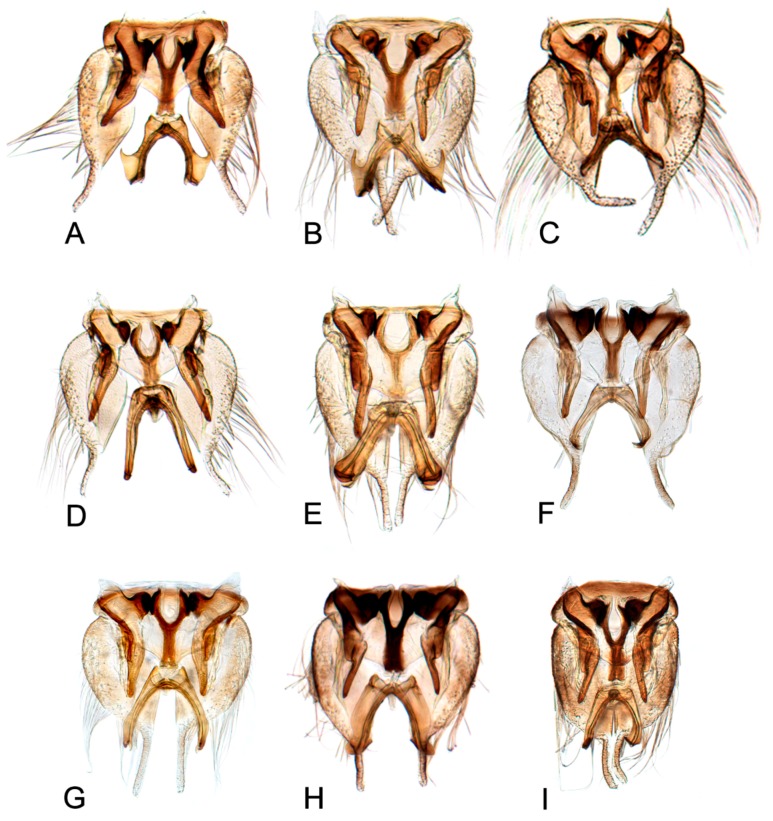
Subgenital plate, valve, style, connective, and aedeagus, dorsal view. (**A**) *H. viraktamathiellus*; (**B**) *H. triangulus*; (**C**) *H. concavus*; (**D**) *H. dietrichi*; (**E**) *H. bucephalus*; (**F**) *H. fletcheri*; (**G**) *H. dichotomous*; (**H**) *H. sellatus*; and (**I**) *H. rectus*.

**Figure 16:**
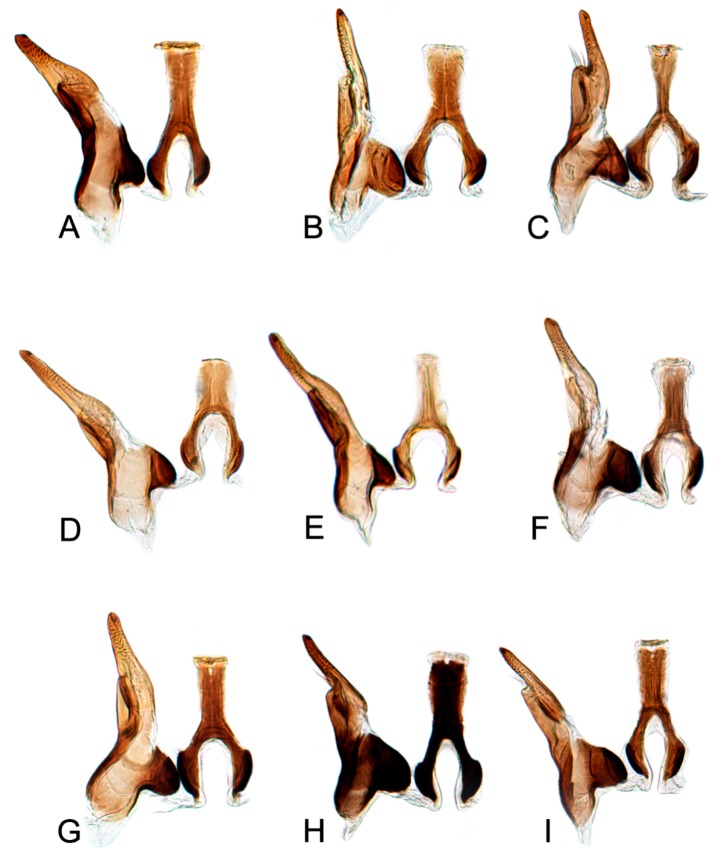
Style and connective, ventral view: (**A**) *H. viraktamathiellus*; (**B**) *H. triangulus*; (**C**) *H. concavus*; (**D**) *H. dietrichi*; (**E**) *H. bucephalus*; (**F**) *H. fletcheri*; (**G**) *H. dichotomous*; (**H**) *H. sellatus*; and (**I**) *H. rectus*.

**Figure 23:**
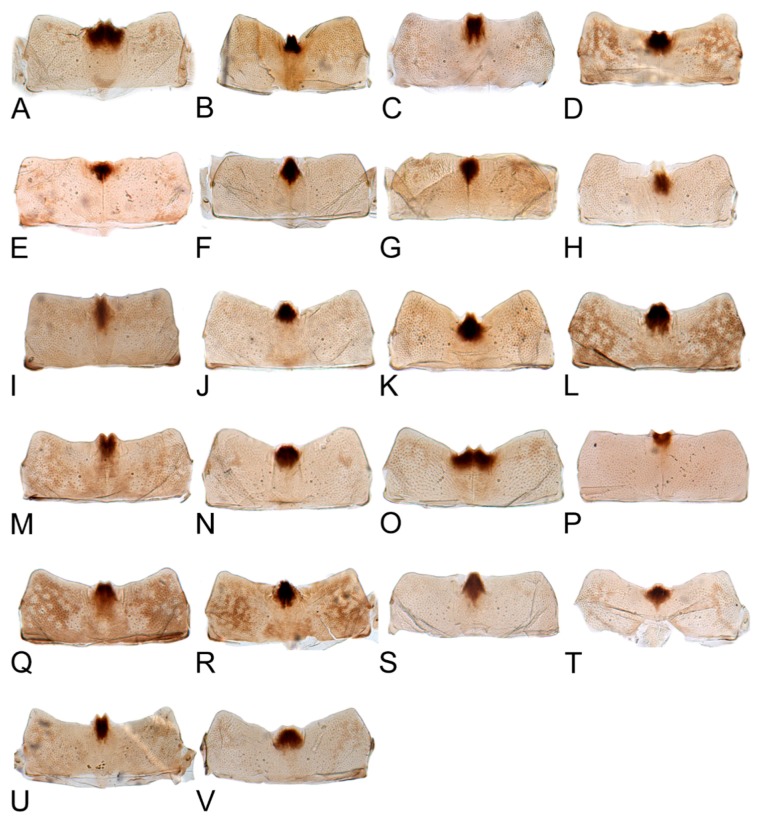
Female sternite VII, ventral view. (**A**) *H. spiniferous*; (**B**) *H. kuohi*; (**C**) *H. tortuosus*; (**D**) *H. aberrans*; (**E**) *H. knightiellus*; (**F**) *H. tenuis*; (**G**) *H. subtilis*; (**H**) *H. expansivus;* (**I**) *H. hamuleus*; (**J**) *H. hamatus*; (**K**) *H. fuscomaculatus*; (**L**) *H. phycitis*; (**M**) *H. paraphycitis*; (**N**) *H. viraktamathiellus*; (**O**) *H. triangulus*; (**P**) *H. concavus*; (**Q**) *H. dietrichi*; (**R**) *H. bucephalus*; (**S**) *H. fletcheri*; (**T**) *H. dichotomous*; (**U**) *H. sellatus*; and (**V**) *H. rectus*.

**Figure 24:**
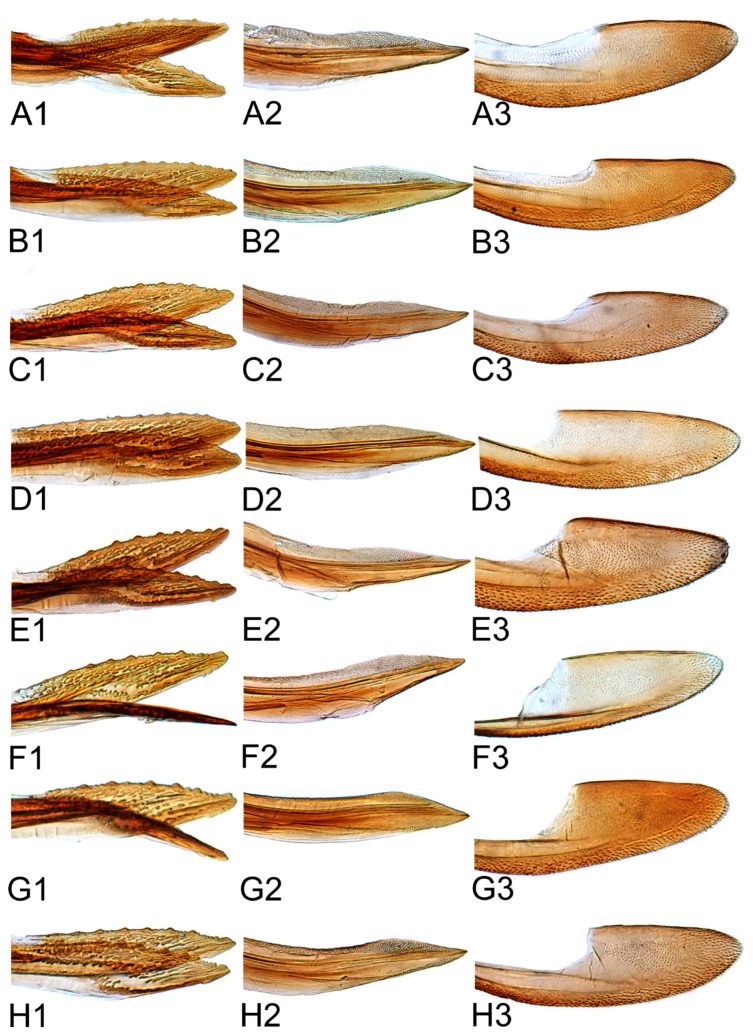
Dissected parts of ovipositor, showing detail of second, first, and third valvulae apex. (**A1**–**A3**) *H. spiniferous*; (**B1**–**B3**) *H. kuohi*; (**C1**–**C3**) *H. tortuosus*; (**D1**–**D3**) *H. aberrans*; (**E1**–**E3**) *H. knightiellus*; (**F1**–**F3**) *H. tenuis*; (**G1**–**G3**) *H. subtilis*; and (**H1**–**H3**) *H. expansivus*.

**Figure 25:**
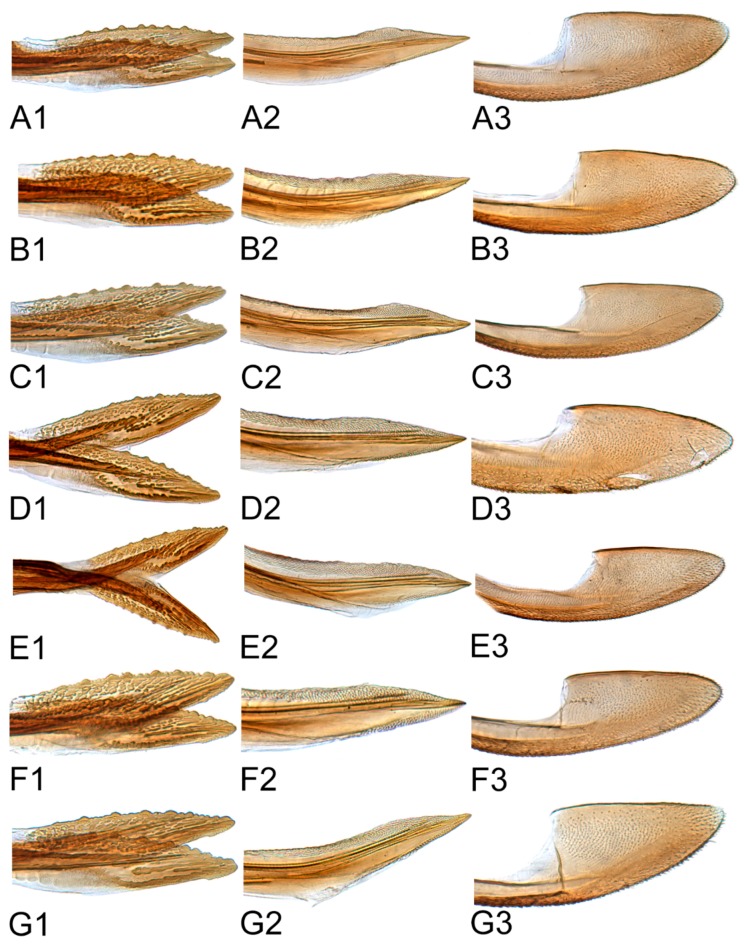
Dissected parts of ovipositor, showing detail of second, first, and third valvulae apex. (**A1**–**A3**) *H. hamuleus*; (**B1**–**B3**) *H. hamatus*; (**C1**–**C3**) *H. fuscomaculatus*; (**D1**–**D3**) *H. phycitis*; (**E1**–**E3**) *H. paraphycitis*; (**F1**–**F3**) *H. viraktamathiellus*; and (**G1**–**G3**) *H. triangulus*.

**Figure 26:**
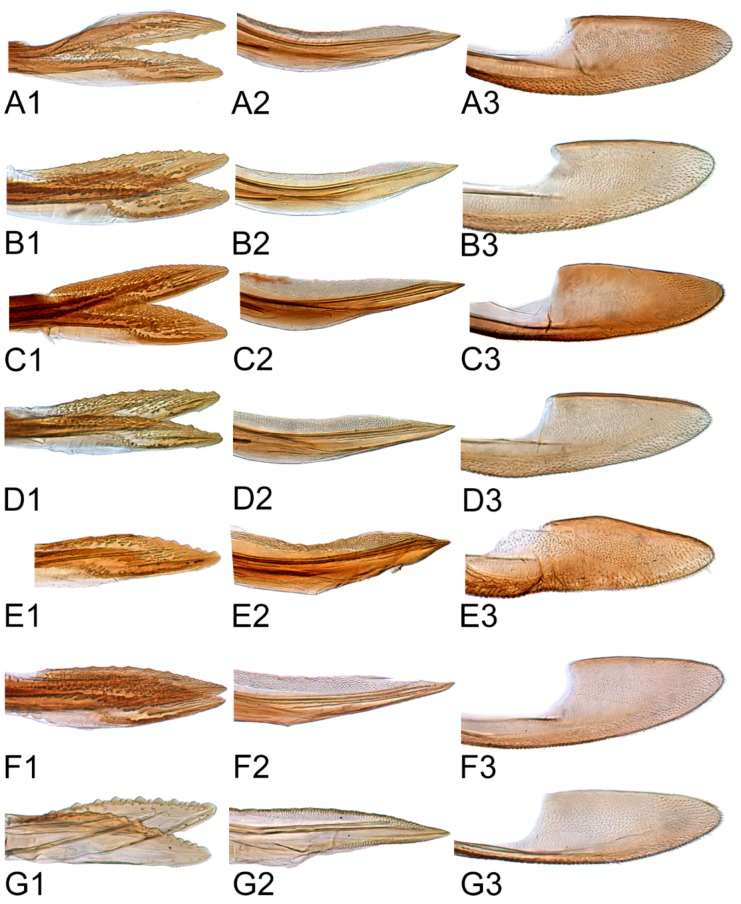
Dissected parts of ovipositor, showing detail of second, first, and third valvulae apex. (**A1**–**A3**) *H. concavus*; (**B1**–**B3**) *H. dietrichi*; (**C1**–**C3**) *H. bucephalus*; (**D1**–**D3**) *H. fletcheri*; (**E1**–**E3**) *H. dichotomous*; (**F1**–**F3**) *H. sellatus*; and (**G1**–**G3**) *H. rectus*.
